# A Pan-Cancer Analysis of the Oncogenic Role of WD Repeat Domain 74 in Multiple Tumors

**DOI:** 10.3389/fgene.2022.860940

**Published:** 2022-04-26

**Authors:** Xiaoxuan Wu, Peng Song, Shun Wang, Zhirong Qian, Jianming Ying, Shugeng Gao, Wenbin Li

**Affiliations:** ^1^ Department of Pathology, National Cancer Center/National Clinical Research Center for Cancer/Cancer Hospital, Chinese Academy of Medical Sciences and Peking Union Medical College, Beijing, China; ^2^ Department of Thoracic Surgical Oncology, National Cancer Center/National Clinical Research Center for Cancer/Cancer Hospital, Chinese Academy of Medical Sciences and Peking Union Medical College, Beijing, China; ^3^ Beidou Academic and Research Center, Beidou Life Science, Guangzhou, China; ^4^ Guangdong Provincial Key Laboratory of Digestive Cancer Research, Scientific Research Center, The Seventh Affiliated Hospital, Sun Yat-sen University, Shenzhen, China; ^5^ Department of Radiation Oncology, Fujian Medical University Union Hospital, Fuzhou, China

**Keywords:** pan-cancer analysis, WD repeat domain 74, oncology, bioinformatics analysis, carcinogenesis

## Abstract

Although emerging patient-derived samples and cellular-based evidence support the relationship between WDR74 (WD Repeat Domain 74) and carcinogenesis in multiple cancers, no systematic pan-cancer analysis is available. Our preliminary research demonstrated that WDR74 is over-expressed in lung squamous cell carcinoma (LUSC) and related with worse survival. We thus investigated the potential oncogenic roles of WDR74 across 33 tumors based on the database of TCGA (The Cancer Genome Atlas) and GEO (Gene Expression Omnibus). WDR74 is highly expressed in most cancers and correlated with poor prognosis in several cancers (all *p* < 0.05). Mutation analysis demonstrated that *WDR74* is frequently mutated in promoter regions of lung cancer. Moreover, we found that CD8^+^ T-cells and the fibroblast infiltration level increased in WDR74 over-expressed cancer cells. The GO (Gene Ontology) enrichment analysis of the WDR74 pathway revealed its participation in cellular biogenesis of the RNA metabolism and its critical role in cancer initiation and progression through the tumor cell energy metabolism. Our first pan-cancer study inferred a relatively comprehensive understanding of the oncogenic roles of WDR74 across various cancers.

## Introduction

Pan-cancer expression analysis plays an important role to figure out the correlation between the gene of interest and its potential mechanisms in carcinogenesis (Barger et al., 2019; Gobin et al., 2019; Cui et al., 2020; Zhang et al., 2020). The publicly funded TCGA (The Cancer Genome Atlas) project and the available GEO (Gene Expression Omnibus) database include functional genomics data sets of different tumors and thus allow us to conduct pan-cancer analysis (Tomczak et al., 2015; Clough and Barrett, 2016; Blum et al., 2018).

*WDR74* is a single copy gene with a conserved protein structure across species (Penel et al., 2009; Smith et al., 1999). WDR74 with six WD40 domains is in the superfamily of WD40 repeat proteins, which have been proved to involve in a wide variety of cellular functions, such as cell division, cell fate determination, gene transcription, transmembrane signaling, mRNA modification, and vesicle fusion (Maserati et al., 2011; Berglund et al., 2008; Weinhold et al., 2014; Lo et al., 2017; Hiraishi et al., 2015; Jin et al., 2020). Our preliminary immunohistochemistry (IHC) analysis based on the tissue microarray (TMA) demonstrated higher expression of WDR74 protein in lung squamous cell carcinoma (LUSC) than in normal tissues (paired *t*-test, *p*-value < 0.001) (Supplementary Figure S1). In addition, *in vitro* studies showed that WDR74 could induce nuclear β-catenin accumulation and activate Wnt responsive genes to promote cancer growth and metastasis in lung and colorectal cancer (Li et al., 2020a; Cai et al., 2021). Moreover, Li et al. claimed that WDR74 modulates tumorigenesis and metastasis through the RPL5-MDM2-p53 pathway in melanoma (Li et al., 2020b).

Although several studies have prompted oncogenic roles of WDR74 in tumors, *WDR74* is not mentioned as a cancer consensus gene in COSMIC (Catalogue of Somatic Mutations in Cancer) (Sondka et al., 2018). Moreover, there is still no pan-cancer analysis on the relationship between WDR74 and various tumor types based on data-driven evidence. Our study, for the first time, based on previous results and combined with the TCGA project and GEO databases, conducted a pan-cancer analysis of WDR74 in various tumors. We also investigated the potential molecular mechanism of WDR74 in the pathogenesis or clinical prognosis of different cancers by analyzing correlative factors to further confirm its oncogenic role in tumors.

## Materials and Methods

### Gene Expression Analysis

To explore WDR74’s function in cancers, we analyze WDR74 expression in different cancers or specific tumor subtypes. Expression profiling was downloaded from TIMER2 (http://timer.cistrome.org/) based on the TCGA project, showing the differences between tumors and adjacent normal tissues of different tumors. Given the lack of the normal issues of several tumors (DLBC, GBMLGG, SKCM, TGCT, THCA), our study combines TCGA with GTEx to conduct the survey through GEPIA2 (http://gepia2.cancer-pku.cn/) (Tang et al., 2019). In addition to the gene expression, the protein expression levels were also obtained from the Ualcan (http://ualcan.path.uab.edu/index.html) (Chen et al., 2019). Furthermore, to determine if WDR74 expression has correlation with tumor staging, we conduct survey on GEPIA2. We logged into the online HPA (Human Protein Atlas) database (https://www.proteinatlas.org/humanproteome/pathology) to obtain the expression data of the WDR74 gene in different cells and tissues under physiological conditions by entering the word “WDR74.” The expression level of the WDR74 protein in a plasma sample was estimated in the HPA database. “Low specificity” was defined by “NX (normalized expression) ≥ 1 in at least one tissue/region/cell type but not elevated in any tissue/region/cell type.”

### Survival Prognosis Analysis

To test the relationship between WDR74 expression and survival prognosis, we used the “Survival Map” module of GEPIA2 to get the OS (overall survival) and DFS (disease-free survival), respectively. Cutoff-high and cutoff-low values were used as the expression thresholds for splitting the high-expression and low-expression cohorts.

Moreover, we obtained archival formalin-fixed paraffin-embedded (FFPE) specimens of 97 LUSC from Cancer Hospital, Chinese Academy of Medical Science, Beijing, China. All the patients have provided informed consent, and the study was approved by the Institutional Review Board of Cancer Hospital, Chinese Academy of Medical Science and Peking Union Medical College. We performed Immunohistochemistry (IHC) on the tissue microarrays according to the protocol of a previous study, with anti-WDR74 antibodies (Santa Cruz Biotechnology, clone E-6, 1:50 dilution).

The score was determined by multiplying the score of staining intensity and the score of positive cells. The detailed rules of scoring the staining intensity were described in supplementary materials and methods. OS of high- and low-WDR74 subgroups of patients was compared using the Kaplan–Meier method with the log-rank test. The data in this part were analyzed and plotted by using the “survival” package of R4.1.0 software (https://www.r-project.org/).

### Genetic Alteration Analysis

Our study used cBioportal (http://cbioportal.org) to analyze the gene alteration based on the TCGA Pan Cancer Atlas Studies tumor by choosing the “cancer type summary” in the query module (Cerami et al., 2012; Gao et al., 2013). Detailed mutated site information of *WDR74* and the 3D structure of WDR74 protein were downloaded by choosing “mutations” in the same module. Moreover, we examined mutation calls from previously published LUSC whole genome sequencing [20 patients from TCGA and 48 patients from pan-cancer analysis of the whole genome (PCAWG) cohort] to analyze WDR74 promoter mutation.

Moreover, 109 patients with LUSC were recruited at the Cancer Hospital, Chinese Academy of Medical Sciences, from December 2017 to December 2019, and all patients provided informed consent. We conducted exome-promoter sequencing on tumor and blood samples to figure out the *WDR74* mutation status in exonic regions. More details can be found in the supplementary material and method.

### Immune Infiltration Analysis

We used TIMER2 to perform immune infiltration analysis. WDR74 immune infiltration was searched in four databases to determine WDR74’s function in the tumor forming. Then, we selected several points with strong correlation to display its scatter plot. The *p*-values and partial correlation (cor) values were obtained *via* the purity-adjusted Spearman’s rank correlation test. We explored the relationship between WDR74 expression and ImmuneScore, StromalScore, and ESTIMATEScore based on R packages “estimate” and analyzed the relationship between WDR74 and immune checkpoints on the website of “http://sangerbox.com/Tool.”

### WDR74-Related Gene Enrichment Analysis

We selected 50 WDR74-binding proteins with experimental support. We set the following main parameters: minimum required interaction score [“Medium confidence (0.400)”], meaning of network edges (“evidence”), max number of interactors to show (“no more than 50 interactors” in the first shell), and active interaction sources (“experiments”). Then, we searched in the “similar gene detection” module to obtain 100 WDR74-correlated targeting genes. The log2 TPM was applied for the dot plot. The *p*-value and the correlation coefficient (R) were indicated. After that, we input these selected genes in TIMER (Tumor Immune Estimation Resource) to get the heatmap in the “Gene_Corr” module.

We use Venn (http://bioinformatics.psb.ugent.be/webtools/Venn/) to get the genes involving in the two parts. The two sets of data were used to perform KEGG (Kyoto Encyclopedia of Genes and Genomes) pathway analysis and GO (Gene Ontology) enrichment analysis by using the R packages “ggplot2” and “clusterProfiler” to conduct GO (Gene Ontology) enrichment analysis. The data for BP (biological process), CC (cellular component), and MF (molecular function) were visualized as bubble charts. Two-tailed *p* < 0.05 was considered statistically significant.

We also conducted gene set enrichment analysis (GSEA) to inspect the statistical significance of a defined set of genes and verify the differences between two biological states. Based on the TCGA database, we divided LUSC samples into two subgroups on the grounds of the median expression level of WDR74. GO gene sets were analyzed by the “clusterProfiler” and “enrichplot” R package to identify functional terms and pathways. Gene set permutations were executed 100 times for each analysis. The criteria of significantly enriched pathways were normalized with *p* < 0.05.

## Results

### WDR74 Expression Analysis Data

Our study first investigated the WDR74 protein structure and the phylogenetic tree data. The genomic location of human WDR74 is shown (Supplementary Figure S2A). The protein structure demonstrated conserved domains among different spices, including the WD40 (cd00200) and WD40 (cl02567) domains (Supplementary Figure S2B). The phylogenetic tree data indicated the evolutionary relationship of the WDR74 protein across different species (Supplementary Figure S3).

Additionally, we explored the expression differences of WDR74 in multiple normal cell lines and tissues. Our results revealed that WDR74 has the highest expression in the bone marrow, followed by the dendritic cells (Supplementary Figure S4A), based on the combination of the HPA (Human Protein Atlas), GTEx, and FANTOM5 (Function Annotation of the Mammalian Genome 5) datasets. Low RNA tissue specificity and low RNA blood cell type specificity were presented (Supplementary Figure S4).

**FIGURE 1:**
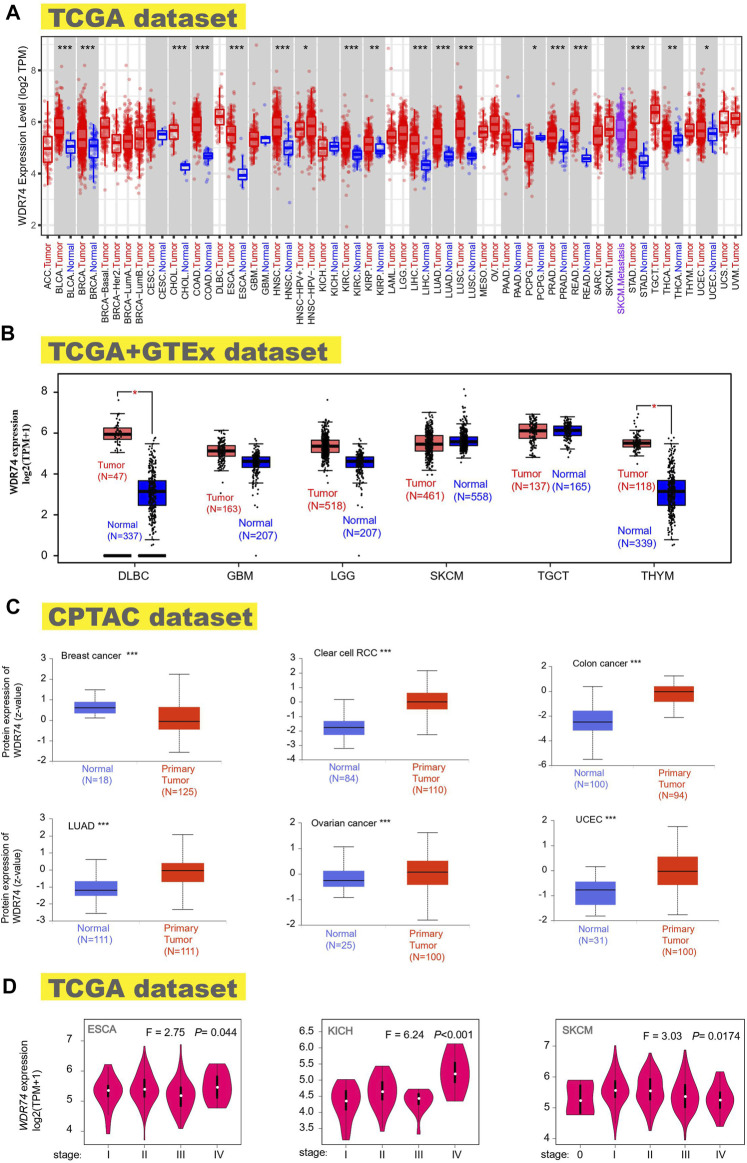
Expression status of the *WDR74* gene in multiple tumors and different pathological stages. We explored the *WDR74* expression difference between tumor samples and paired normal tissues in different cancers or specific cancers utilizing TIMER2. *p < 0.05; **p < 0.01; ***p < 0.001 **(A)**. Combined with normal tissues in the GTEx database, *WDR74* expression levels were analyzed for several tumors (DLBC, GBM, LGG, SKCM, TGCT, and THYM) in the TCGA project. *p < 0.05 **(B)**. WDR74 total protein expressions of normal tissues and primary tissues were obtained from the CPTAC dataset in breast cancer, clear cell RCC, colon cancer, LUAD, ovarian cancer, and UCEC. ***p < 0.001 **(C)**. *WDR74* expression levels of different pathological stages were figured out in ESCA, KICH, and SKCM based on TCGA data **(D)**.

Gene expression analysis was conducted in the TCGA cancers, showing the significant difference between normal tissues and tumor tissues in the BLCA (bladder urothelial carcinoma), BRCA (breast invasive carcinoma), CHOL (cholangiocarcinoma), COAD (colon adenocarcinoma), ESCA (esophageal carcinoma), HNSC (head and neck squamous cell carcinoma), KIRC (kidney renal clear cell carcinoma), LIHC (liver hepatocellular carcinoma), LUAD (lung adenocarcinoma), LUSC, PRAD (prostate adenocarcinoma), READ (rectum adenocarcinoma), STAD (stomach adenocarcinoma) (*p* < 0.001), THCA (thyroid carcinoma), KIRP (kidney renal papillary cell carcinoma) (*p* < 0.01), PCPG (pheochromocytoma and paraganglioma), and UCEC (uterine corpus endometrial carcinoma) (*p* < 0.05) (Figure 1A). However, several tumor samples lack normal tissues as controls in the TCGA database, including DLBC, GBM, LGG, SKCM, TGCT, and THYM. To explore the expression feature in these tumors, we combined the TCGA and GTEx datasets to get enough normal samples as paired tissues and observed the high-expression WDR74 in DLBC (lymphoid neoplasm diffuse large B-cell lymphoma) and THYM (thymoma) (*p* < 0.05) (Figure 1B).

The analysis results of the CPTAC dataset showed higher expression of WDR74 protein in breast cancer, clear cell RCC, colon cancer, LUAD, ovarian cancer, and UCEC (*p* < 0.001) (Figure 1C). The pooling analysis results in the Oncomine database further demonstrated that WDR74 is highly expressed in brain and CNS cancer, breast cancer, colorectal cancer, lung cancer, or lymphoma compared with normal controls (Supplementary Figure S6). The “Pathological Stage Plot” module of the HEPIA2 database was applied to depict the correlation between WDR74 expression and the pathological stages of cancers, including ESCA, KICH (Kidney Chromophobe), and SKCM (Skin Cutaneous Melanoma) (Figure 1D, all *p* < 0.05).

### Survival Analysis Data

**FIGURE 2:**
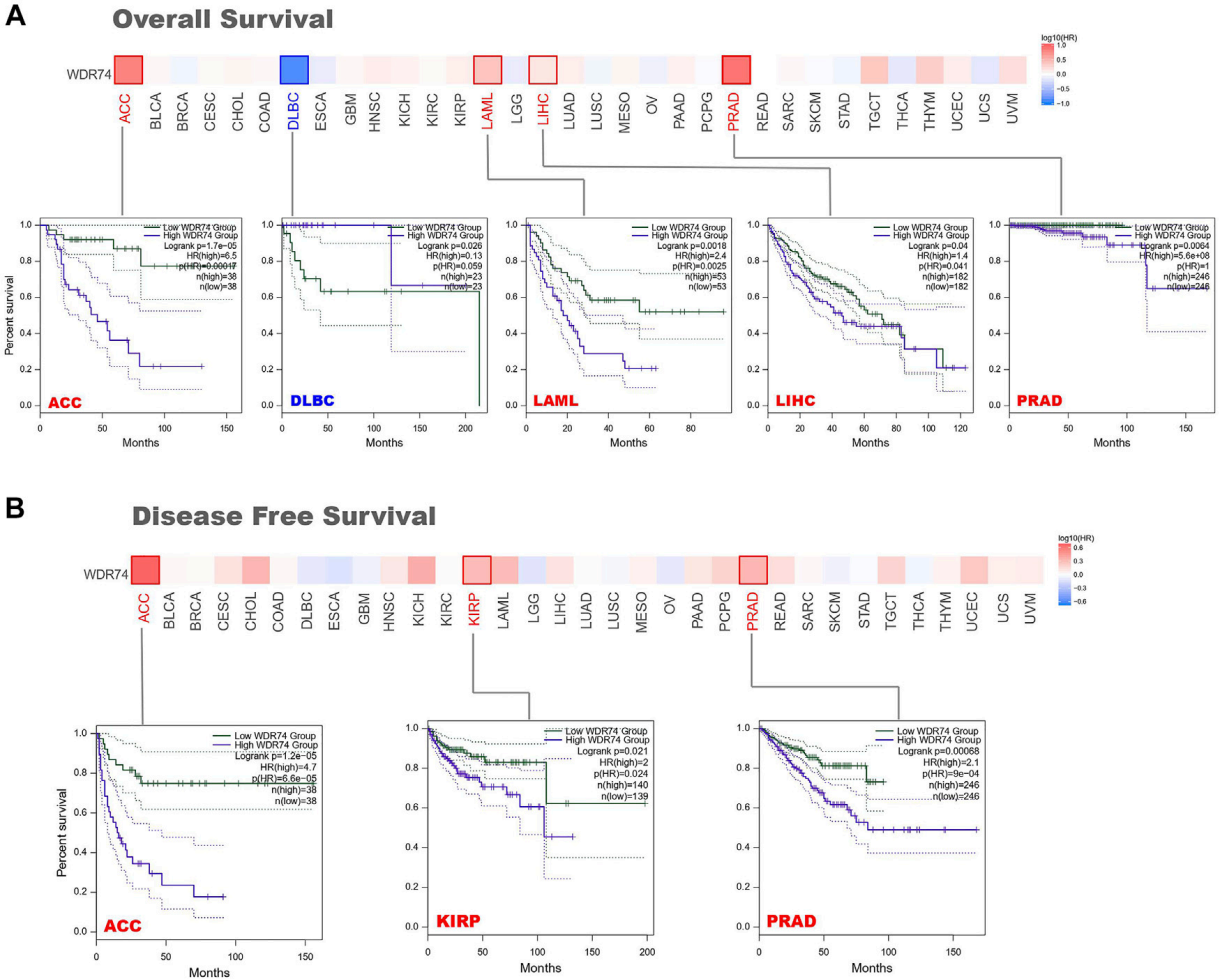
Survival prognosis between groups with high and low WDR74 expression levels was obtained on GEPIA2 based on the TCGA project. **(A)** The Kaplan-Meier analysis of overall survival with WDR74 expression in the ACC, DLBC, LAML, LIHC and PRAD. **(B)** The Kaplan–Meier analysis of disease-free survival with WDR74 expression in the ACC, KIRP and PRAD.

Divided by the expression levels of WDR74, patients’ prognosis was analyzed based on the datasets of TCGA and GEO. Highly expressed WDR74 was associated with poor prognosis of OS in cancers of ACC (adrenocortical carcinoma) (*p* < 0.001), LAML (acute myeloid leukemia) (*p* < 0.01), LIHC (*p* < 0.05), and PRAD (*p* < 0.01) from the TCGA project (Figure 2A). It is worth noting that in DLBC, WDR74 expression increased, but the prognosis was good, which was contrary to other cancers. It may be limited by the sample sizes that were no more than 50 in high and low expression groups. Moreover, the oncogenic role of WDR74 and the underlying molecular mechanism need more in-depth evidence to identify. As for DFS, there was a similar connection between high WDR74 expression and poor prognosis within the TCGA cases of ACC (*p* < 0.001), KIRP (*p* < 0.05), and PRAD (*p* < 0.001) (Figure 2B).

Furthermore, we utilized the Kaplan–Meier plotter tool to obtain the correlation between WDR74 expression and prognosis in breast cancer, lung cancer, gastric cancer, ovarian cancer, and liver cancer (Supplementary Figures S7, S8). We observed that WDR74 expression was negatively correlated with the OS (*p* < 0.001) and DMFS (*p* < 0.01) for breast cancer. Additionally, we found that the high WDR74 expression level predicted the poor OS, FP, and PPS (all *p* < 0.001) in lung cancer. However, a low expression level of WDR74 was associated with poor OS (*p* < 0.01) and PFS (*p* < 0.05) in ovarian cancer and poor OS (*p* = 0.01) and FP (*p* < 0.05) in gastric cancer. Our analysis did not show a significant differential prognosis in OS, PFS, RFS, and DSS prognosis of liver cancer. Moreover, subgroup analyses of these cancers were conducted to investigate the distinct conclusions (Supplementary Tables S1–S5).

In summary, WDR74 expression level was significantly associated with the poor prognosis in several cancers, especially in lung cancer, ACC, and PRAD.

### Genetic Alteration Analysis Data

**FIGURE 3:**
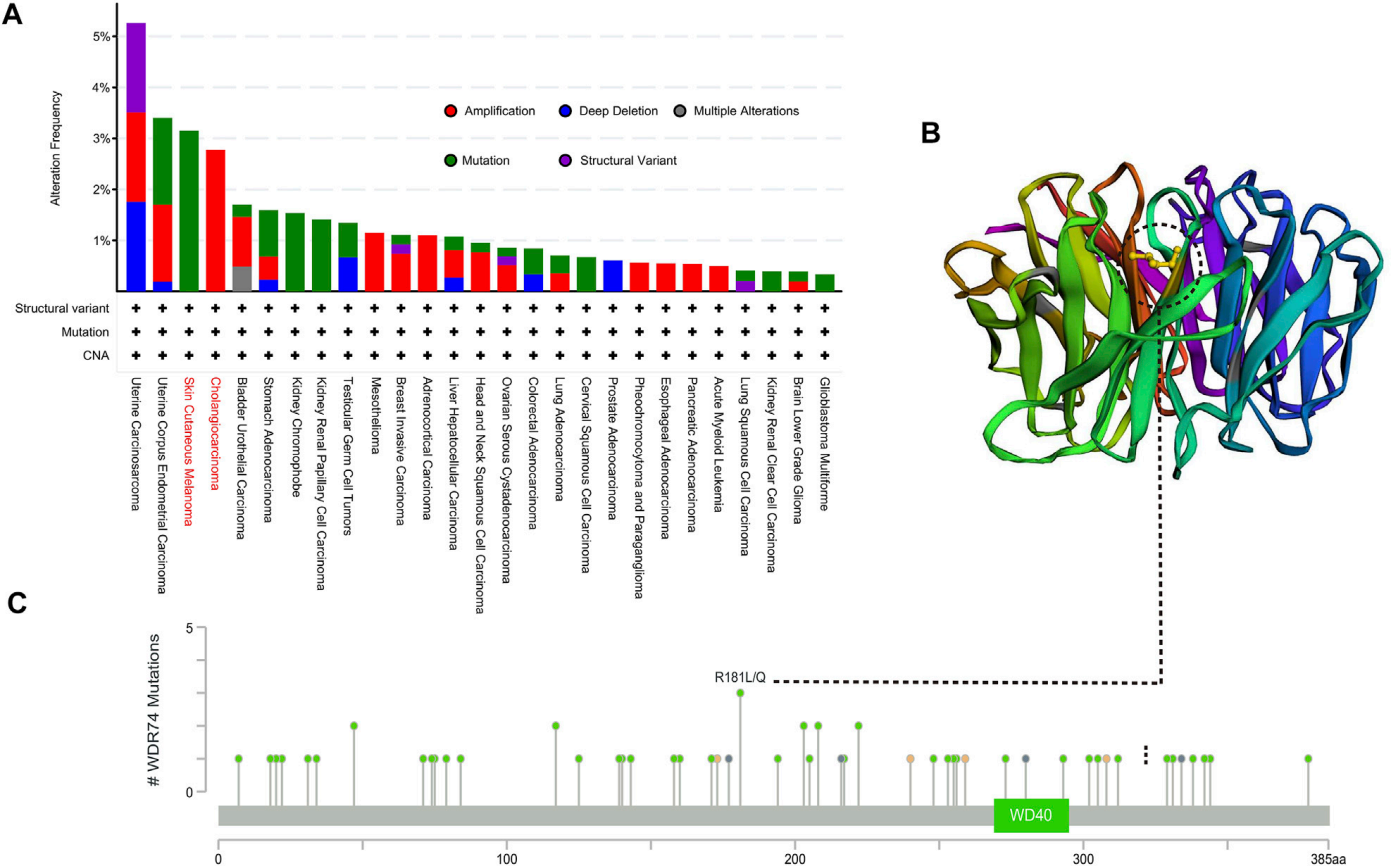
Mutation features of *WDR74* in multiple tumors based on TCGA were analyzed by the cBioPortal tool. The alteration frequency of mutation types **(A)** and mutation sites located on the coding region **(C)** are shown. The mutation site with the highest alteration frequency (R181L/Q) was highlighted in the 3D structure of WDR74 **(B)**.

Based on our previous conclusion, WDR74 was highly expressed in multiple tumor tissues and correlated with worse prognosis in several tumors, suggesting its potential oncogenic role in carcinogenesis. Therefore, we next explored the genetic variations of *WDR74*. We found that uterine carcinosarcoma patients bear the highest genetic alteration frequency (>5%) of WDR74. The “mutation” type and “amplification” type account for a significant portion of the alteration in the skin cutaneous melanoma and cholangiocarcinoma, respectively. We further explored the types, sites, and case number of *WDR74* genetic alteration, which are exhibited in Figure 3A. It is noted that the R181L/Q missense mutation was verified in two cases of skin cutaneous melanoma and one case of glioblastoma, suggesting its potential role as a hotspot mutation site. The R181 site was highlighted in the 3D structure (Figure 3C). Except the coding regions, we also analyzed the functional promoter regions of WDR74.

We found 14.7% (16/109) cases with promoter alterations in a ∼1-kb window near the 5′ end of the *WDR74* gene, most of which clustered at the start of the 5′ flank region, including single nucleotide variations, small insertions, and deletions. We examined mutation calls from previously published LUSC whole genome sequencing [20 patients from TCGA and 48 patients from pan-cancer analysis of the whole genome (PCAWG) cohort]. The analysis showed that three out of 20 cases from the TCGA dataset had *WDR74* promoter mutations within the range of chr11. 62841736 to chr11. 62841875 and of chr11. 62841450 to chr11. 62841654. (Supplementary Table S6). Moreover, the methylation analysis of the promoter region showed the negative association between expression and *WDR74* methylation in LUSC (Supplementary Figure S9).

### Immune Infiltration Analysis Data

Tumor-infiltrating immune cells which comprise a large proportion of cells in the tumor microenvironment were correlated with the initiation, progression, and metastasis of cancer (Fridman et al., 2011; Steven and Seliger, 2018). Cancer-associated fibroblasts (CAFs) are the most abundant and are critically involved in cancer progression in the tumor microenvironment. CAFs affect cancer initiation and development by regulating the biology of tumor cells and other stromal cells *via* cell–cell contact, releasing numerous regulatory factors and synthesizing and remodeling the extracellular matrix (Chen and Song, 2019).

Based on the TCGA project, the study evaluated the potential connection between the infiltration level of different immune cells and WDR74 gene expression applying the TIMER, CIBERSORT, QUANTISEQ, TIDE (Tumor Immune Dysfunction and Exclusion), XCELL, MCPCOUNTER, and EPIC algorithms.

**FIGURE 4:**
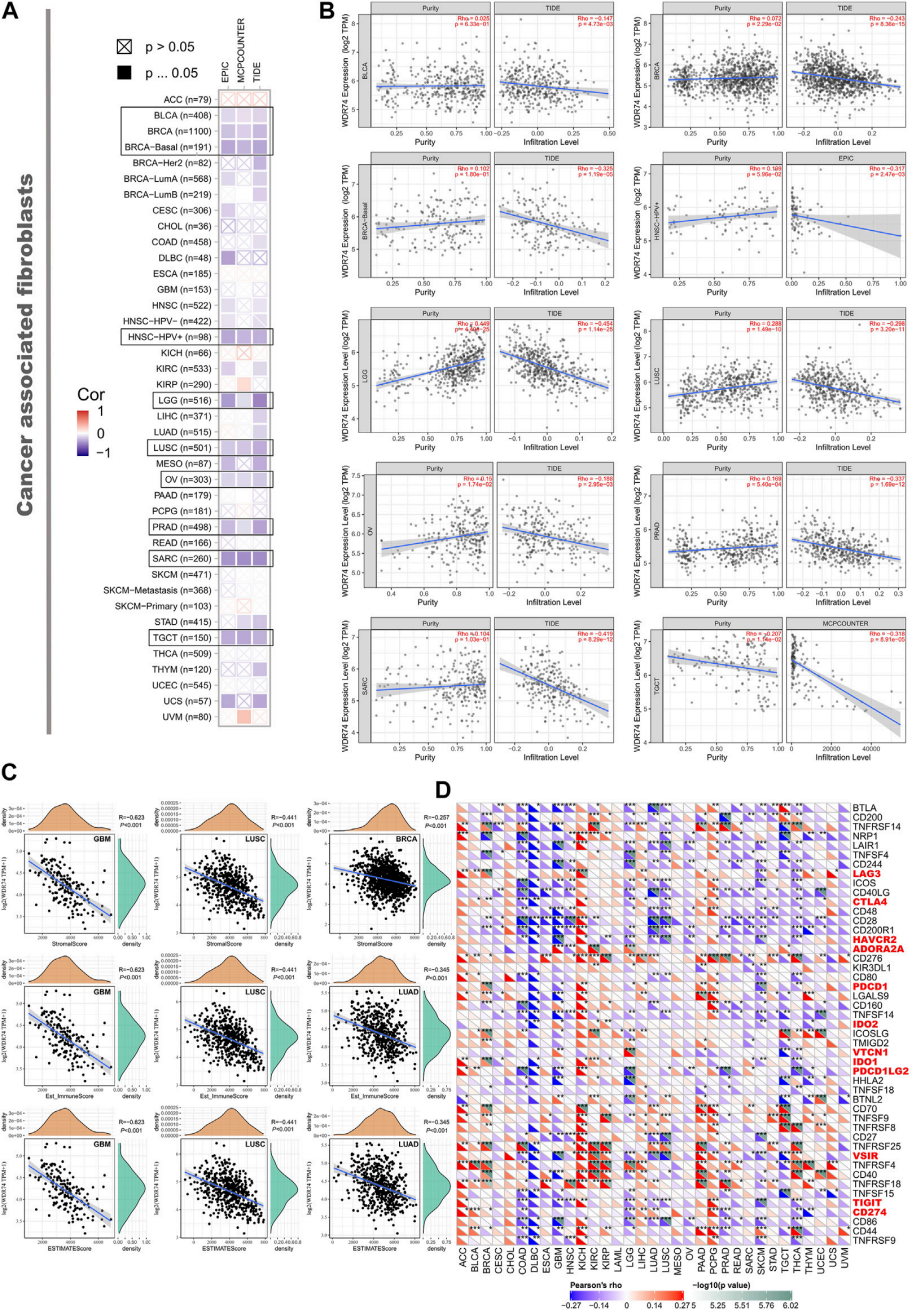
We applied different algorithms to figure out the potential correlation between the expression level of the *WDR74* gene and the infiltration level of cancer-associated fibroblasts in TCGA cancers **(A)**. The correlation between *WDR74* expression and the estimated infiltration value of cancer-associated fibroblasts is shown as a scatterplot in BLCA, PRAD, SARC, and LUSC **(B)**. The correlation analysis of ImmuneScore, StromalScore, and EstimateScore with WDR74 expression in GBM, LUSC, BRCA, and LUAD is shown as a scatterplot **(C)**. We also explored correlation between WDR74 expression and immune-related cytokines and immune checkpoint expression across 33 TCGA tumors **(D)**.

The statistical results revealed that the immune infiltration of CD8^+^ T-cells was correlated with WDR74 expression positively in LIHC and negatively in SKCM-Metastasis (Supplementary Figure S10). Meanwhile, WDR74 expression was negatively associated with the estimated infiltration value of cancer-associated fibroblasts for the TCGA tumors of BLCA, BRCA, HNSC, LGG, LUSC, OV, PRAD, SARC, and TGCT, visualizing as scatterplots (Figure 4B). For instance, we observed significant negative correlation between the WDR74 expression level in SARC and the infiltration level of cancer-associated fibroblasts (cor = −0.419, *P* < 0.001) utilizing the TIDE algorithm. ImmuneScore, StromalScore, and EstimateScore are standardized measurements of immune cells and stromal cells in defined regions of the tumor, which were estimation indexes of tumor purity and proved to be predictive prognostic factors independent of other clinical features in several cancers. Tumor microenvironment analysis revealed that ImmuneScore, StromalScore, and EstimateScore were correlated with a low WDR74 expression level in LUSC (R = −0.441, *p* < 0.001) (Figure 4C).

Moreover, high-expression WDR74 correlated with expression of immune checkpoints VSIR, HAVCR2, and TIGIT (all *p* < 0.001) in LUSC, suggesting that WDR74 is a potential target in immune therapy (Figure 4D).

### Enrichment Analysis of WDR74-Related Partners

**FIGURE 5:**
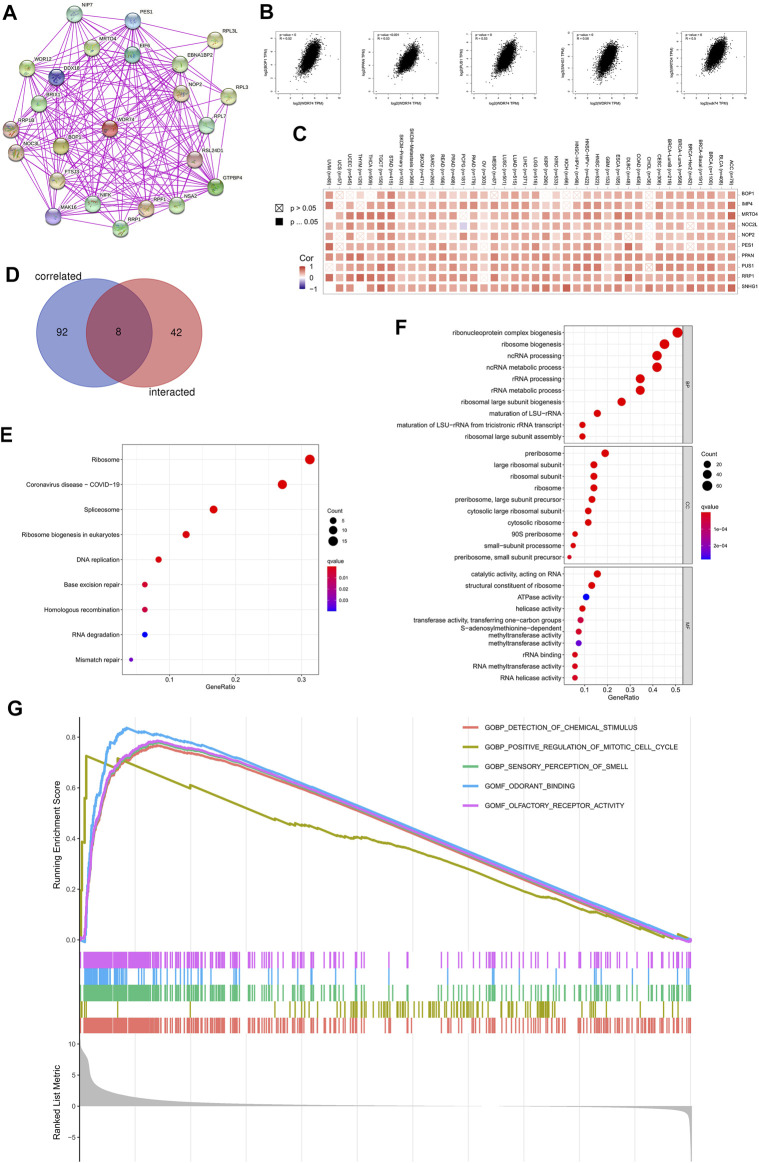
Enrichment analysis on *WDR74* were conducted to further investigate its role in tumorigenesis. We obtained WDR74-binding proteins with experimental evidence using the STRING tool **(A)**. Top 100 WDR74-correlated genes were selected from TCGA projects and the correlation between WDR74 expression and selected targeting genes, including BOP1, PPAN, PUS1, SNHG1, and MRTO4, was analyzed on GEPIA2 **(B)**. The corresponding heatmap data in the detailed cancer types are shown **(C)**. We conducted an intersection analysis between the selected 50 WDR74-binding genes and 100 WDR74-correlated genes **(D)**. We utilized WDR74-binding genes and WDR74-interacted genes to perform KEGG pathway analysis **(E)**. The bubble chart of GO analysis is shown **(F)**. Based on GSEA, GO enrichment analysis on LUSC is shown **(G)**.

We selected the targeting WDR74-binding proteins and the WDR74 expression-correlated genes to conduct pathway enrichment analyses. We used STRING to collect 50 WDR74-binding proteins which were endorsed by experimental evidence and depicted the relationship with a high confidence (interaction score >0.700) between them in the forms of the network (Figure 5A).

Additionally, we sought out top 100 genes that correlated with WDR74 by combing all TCGA tumor data in GEPIA2. As shown in Figure 5B, the WDR74 expression level was positively correlated with that of BOP1 (R = 0.52), PPAN (R = 0.53), PUS1 (R = 0.53), SNHG1(R = 0.56), and MRTO4 (calumenin) (R = 0.5) genes (all *p* < 0.001). The corresponding heatmap data presented a positive correlation between WDR74 and the above genes in the majority of detailed cancer types (Figure 5C). Moreover, we conduct an intersection analysis of the WDR74-binding and correlated genes (Figure 5D).

The KEGG analyses suggested that “Ribosome” might be involved in the effect of WDR74 on tumor pathogenesis (Figure 5E). The GO enrichment analysis data showed that most of these genes were linked to the pathways or cellular biology of the RNA metabolism, such as ribonucleoprotein complex biogenesis, ribosome biogenesis, and ncRNA processing (Figure 5F). Based on GSEA, GO enrichment analysis revealed that WDR74-related genes were involved in the process of the cell cycle in the LUSC (Figure 5G).

## Discussion

In the study, we conducted expression analysis, survival analysis, genetic alteration analysis, immune infiltration analysis, and enrichment analysis of WDR74 to explore its potential role in carcinogenesis. Our pan-cancer analysis showed high-level expression of WDR74 in tumor tissues (*p* < 0.05) and its correlation with poor prognosis in several tumors. Mutation analysis demonstrated that *WDR74* is frequently mutated in promoter regions of lung cancer and has the R181L/Q hotspot mutation in skin cutaneous melanoma and glioblastoma. Previous researchers claimed that WDR74 promotes proliferation and metastasis in lung and colorectal cancer cells through regulating the Wnt/β-catenin signaling pathway (Liu et al., 2018). It has also been proved that WDR74 modulates melanoma cell proliferation and metastasis through the RPL5-MDM2-p53 pathway (Li et al., 2020b). Based on a pan-cancer pathway enrichment analysis, we first identified that WDR74 participates in cellular biogenesis of the RNA metabolism and its critical role in cancer initiation and progression through the tumor cell energy metabolism.

It is well known that hyperactivation ribosome biogenesis which can be initiated by oncogenes or the loss of tumor suppressor genes has causal associations with elevated cancer risk (Penzo et al., 2019). Mutations in genes encoding ribosomal proteins (RPs) or ribosome assembly factors play a critical role in cancer initiation and progression. For example, the expression of the truncated ΔN-netrin-1 (nucleolar N-terminal truncated isoform of netrin 1) was found to drive rDNA transcription and pre-rRNA processing and to increase the number of mature ribosomes in tumor cells, promoting the malignant phenotype (Delloye-Bourgeois et al., 2012). This process was reported to involve protein kinase Cι type (PKCι)-mediated phosphorylation of the epithelial cell-transforming sequence 2 oncogene (ECT2) and the subsequent binding of ECT2 to the nucleolar transcription factor upstream-binding factor 1 (UBF1), which promotes rDNA transcription (Pelletier et al., 2018). Similarly, WDR74 participates in the early cleavage of pre-rRNA processing and regulates appropriate maturation of the pre-60S particles (Hiraishi et al., 2018; Ishida et al., 2021). Our GO enrichment analysis revealed that WDR74-binding components and WDR74 expression-related genes were strongly correlated to the pathways or cellular biology of the RNA metabolism, such as ribonucleoprotein complex biogenesis, ribosome biogenesis, and ncRNA processing, to promote tumorigenesis. Our study provided some evidence supporting these feasible molecular mechanisms, and more efforts are needed to identify them in tumors.

Previous research reported that the *WDR74* promoter is frequently mutated in various cancers (ICGC/TCGA Pan-Cancer Analysis of Whole Genomes Consortium, 2020). In particular, the 5’ untranslated region (UTR) and promoter of WDR 74 were found to be under purifying selection and highly enriched for mutations, and these mutations were broadly distributed across numerous positions. In addition, like our study, the coding sequences of WDR74 did not contain any mutations in LUAD and LUSC tumors. Khurana et al. also reported WDR74 promoter mutations in two of the 20 prostate cancer genomes, suggesting its widely mutated characteristic in multiple tumors. Mutations in non-coding regions, such as UTR and the promoter, usually generated non-functional single-nucleotide polymorphism. However, recent studies have established a comprehensive method to identify important driver mutations in non-coding regions and implicate new driver genes in the development of specific cancers. Among them, *TERT* promoter mutations are particularly common in various cancers. *TERT* can be transcriptionally activated by somatic point mutations in the promoter, which introduce *de novo* transcription factor binding sites and accelerated gene over-expression. In melanoma, glioma, hepatocellular carcinoma, urothelial carcinoma, and others, *TERT* promoter mutations have been found to define subsets of patients with adverse disease outcomes (Heidenreich and Kumar, 2017).

Collectively, WDR74 is overexpressed in multiple tumors and correlated with worse survival prognosis. In addition, WDR74 participates in tumorigenesis through the tumor cell energy metabolism pathway and holds the potential role as a diagnostic biomarker as well as future therapeutic targets in multiple tumors.

## Data Availability

The original contributions presented in the study are included in the article/Supplementary Material, further inquiries can be directed to the corresponding authors.

## Abbreviations

ACC, adrenocortical carcinoma; BLCA, bladder urothelial carcinoma; BRCA, breast invasive carcinoma; CHOL, cholangiocarcinoma; COAD, colon adenocarcinoma; DLBC, lymphoid neoplasm diffuse large B-cell lymphoma; ESCA, esophageal carcinoma; GEO, gene expression omnibus; GO, gene ontology; HNSC, head and neck squamous cell carcinoma; KEGG, Kyoto Encyclopedia of Genes and Genomes; KICH, kidney chromophobe; KIRC, kidney renal clear cell carcinoma; KIRP, kidney renal papillary cell carcinoma; LAML, acute myeloid leukemia; LIHC, liver hepatocellular carcinoma; LUAD, lung adenocarcinoma; LUSC, lung squamous cell carcinoma; OS, overall survival; PCPG, pheochromocytoma and paraganglioma; PRAD, prostate adenocarcinoma; READ, rectum adenocarcinoma; SKCM, skin cutaneous melanoma; STAD, stomach adenocarcinoma; TCGA, The Cancer Genome Atlas; THCA, thyroid carcinoma; UCEC, uterine corpus endometrial carcinoma; WDR74, WD Repeat Domain 74.
