# Supplementary material for: A Pan-Cancer Analysis of the Oncogenic Role of WD Repeat Domain 74 in Multiple Tumors
Source: Front Genet. 2022 Apr 26;13:860940. doi: 10.3389/fgene.2022.860940 (PMC9086290; doi:10.3389/fgene.2022.860940)
Supplement: Supplementary file 1 [file DataSheet3.docx]

Supplementary materials and methods

**Immunohistochemistry analysis**

We obtained archival formalin-fixed paraffin-embedded (FFPE) specimens of 97 LUSC from Cancer Hospital, Chinese Academy of Medical Science, Beijing, China. We performed Immunohistochemistry (IHC) on the tissue microarray arrays according to the protocol of a previous study, with anti-WDR74 antibody (Santa Cruz Biotechnology, clone E-6, 1:50 dilution). This study was approved by our institutional review board of Cancer Hospital, Chinese Academy of Medical Science.

The score was determined by multiplying the score of staining intensity and score of positive cells. The score of staining intensity was scored as follows: 0 for no staining, 1 for weak staining, 2 for moderate staining and, 3 for strong staining. The score for percentage of positive area was kept in the range of 1 to 4, based on: less than 5% positive cells for 0; 5-25% positive cells for 1; 26–50% positive cells for 2; 51-75% positive cells for 3; greater than 75% positive cells for 4. Further, for statistical analyses the scores of 0-1 were treated as low expression, scores of 2-3 as high expression. OS of high- and low-WDR74 subgroups of patients was compared using the Kaplan-Meier method with the log-rank test. The data in this part were analyzed and plotted by using the "survival" package of R4.1.0 software (https://www.r-project.org/).

**Gene mapping and protein structure analysis**

The WDR74 genome location information was downloaded from UCSC Genome Browser (http://genome.ucsc.edu/). WDR74 conserved functional domain analysis was obtained on the "HomoloGene" module of NCBI (National Center for Biotechnology Information) (https://www.ncbi.nlm.nih.gov/homologene/). We utilized the MEGA (Molecular Evolution Genetics Analysis) software to conduct phylogenetic tree analysis of WDR74.

**Gene expression analysis of HPA**

We first logged into the online HPA (Human protein atlas) database (https://www.proteinatlas.org/humanproteome/pathology) and obtained the expression data of the WDR74 gene in different cells and tissues under physiological conditions by entering the word "WDR74”. The expression level of the WDR74 protein in a plasma sample was estimated in the HPA database. “Low specificity” was defined by “NX (Normalized expression) ≥ 1 in at least one tissue/region/cell type but not elevated in any tissue/region/cell type”.

**Gene expression analysis of Oncomine**

We got the expression difference data of the *WDR74* gene between tumor tissues and normal tissues based on Oncomine database ([https://www.oncomine.org/resource/main.html](https://www.proteinatlas.org/humanproteome/pathology)). The threshold was set as P-value =0.05, fold change =1.5. The median rank for WDR74 across each of the analyses, the P-value for the median-ranked analysis, and the legends of the enrolled studies were supplied.

**Survival prognosis analysis of Kaplan-Meier plotter**

We obtained the OS, DMFS (distant metastasis-free survival), RFS (relapse-free survival), PPS (post-progression survival), FP (first progression), DSS (disease-specific survival), and PFS (progress-free survival) analysis based on different GEO datasets on the interactive operation interface of the Kaplan-Meier plotter (http://kmplot.com/analysis/). We set the threshold as “autoselect best cutoff” to divide the cases of lung, ovarian, lung, gastric, and liver cancers into two groups. The hazard ratio (HR), 95% confidence intervals and log-rank P-value were computed, and the Kaplan-Meier survival plots were generated. We used “forestplot” R package to pool the above survival data of WDR74. Moreover, we filtered the clinical factors [e.g., histology, gender, smoking history, stage, grade, surgery, radiotherapy, or chemotherapy, etc.] for a series of subgroup analyses.

**DNA methylation analysis**

The DNA methylation level analysis was downloaded from the MEXPRESS web (<https://mexpress.be/>) across TCGA tumors. The beta value of each sample and the Benjamini-Hochberg-adjusted P-value and Pearson correlation coefficient (R) value were obtained.

***WDR74* mutation analysis in LUSC**

Patient collection and clinical data：The cohort consisted of samples from 109 patients with lung squamous cell carcinoma were collected at the Cancer Hospital, Chinese Academy of Medical Sciences from December 2017 to December 2019 after informed consent. Patients were selected only if their treatment plan required surgical resection, pathologically confirmed lung squamous cell carcinoma, with complete medical records and had received no prior treatment for their disease, including chemotherapy or radiotherapy or immunotherapy. DNA were extracted from tumor and adjacent normal tissue specimens using a modification of the DNA/RNA AllPrep kit (Qiagen). DNA was extracted from blood using the QiaAmp DNA Blood Midi kit (Qiagen). This study was approved by our institutional review board and ethics committee of Cancer Hospital, Chinese Academy of Medical Science.

Exome-promoter discovery library and target gene panel library preparation and sequencing：Custom whole-exome promoter capture was performed using SeqCap EZ MedExome Plus configuration. This extended human content was manufactured by Roche NimbleGen (Roche, USA), with up to ~148Mb of capture regions added to the existing MedExome design with the SeqCap EZ MedExome Plus configuration. The extended whole-exome promoter configuration includes the standard exome targets and the extra 3kb promoter regions for each human gene. Hereby a promoter region was defined as 1.5kb up- and downstream away from the TSS (Transcript Start Site) for a gene’s primary transcript. We also constructed a custom targeted gene capture panel for validation. The custom capture panel was based on SeqCap EZ Choice probes library which was also manufactured by Roche, with ~2.03Mb capture target regions of 450 gene whole exons, 70 gene promoter regions and 22 gene intronic regions.

In brief, 3 µg of human genomic DNA was sheared on BioRuptor DNA Fragmentation System to the average DNA fragment size of 200 bp, followed by end-repair, ligated with barcoded Illumina sequencing adapters, amplified, size selected, and subjected to in-solution hybrid capture using the Exome Plus configuration set or custom targeted gene capture panel. Resulting Illumina sequencing libraries were then qPCR quantified, pooled, and sequenced with 150 base-paired-end reads using Illumina NovaSeq 6000 System sequencers (Illumina, USA), to produce approximately 150x and 50x coverage data for per tumor exome and blood exome or normal exome respectively.

SNVs, short indels and CNVs calling：For somatic SNVs (single nucleotide variation) and short indels calling we processed the raw fastq data according GATK Data pre-processing for variant discovery pipeline and somatic SNV and short indels was identified by Strelka2. For germline SNVs and indels the raw data was aligned by minimap2 and called by DeepVariant. No further filtration was applied to the calling results. All variants were annotated by ANNOVAR. For CNVs (copy number variant) detection we first calculated the sample segment allele counts by GATK somatic CNV pipeline and copy number was finally estimated by GISTIC2.
