# Supplementary figures and images for: A Pan-Cancer Analysis of the Oncogenic Role of WD Repeat Domain 74 in Multiple Tumors
Source: Front Genet. 2022 Apr 26;13:860940. doi: 10.3389/fgene.2022.860940 (PMC9086290; doi:10.3389/fgene.2022.860940)

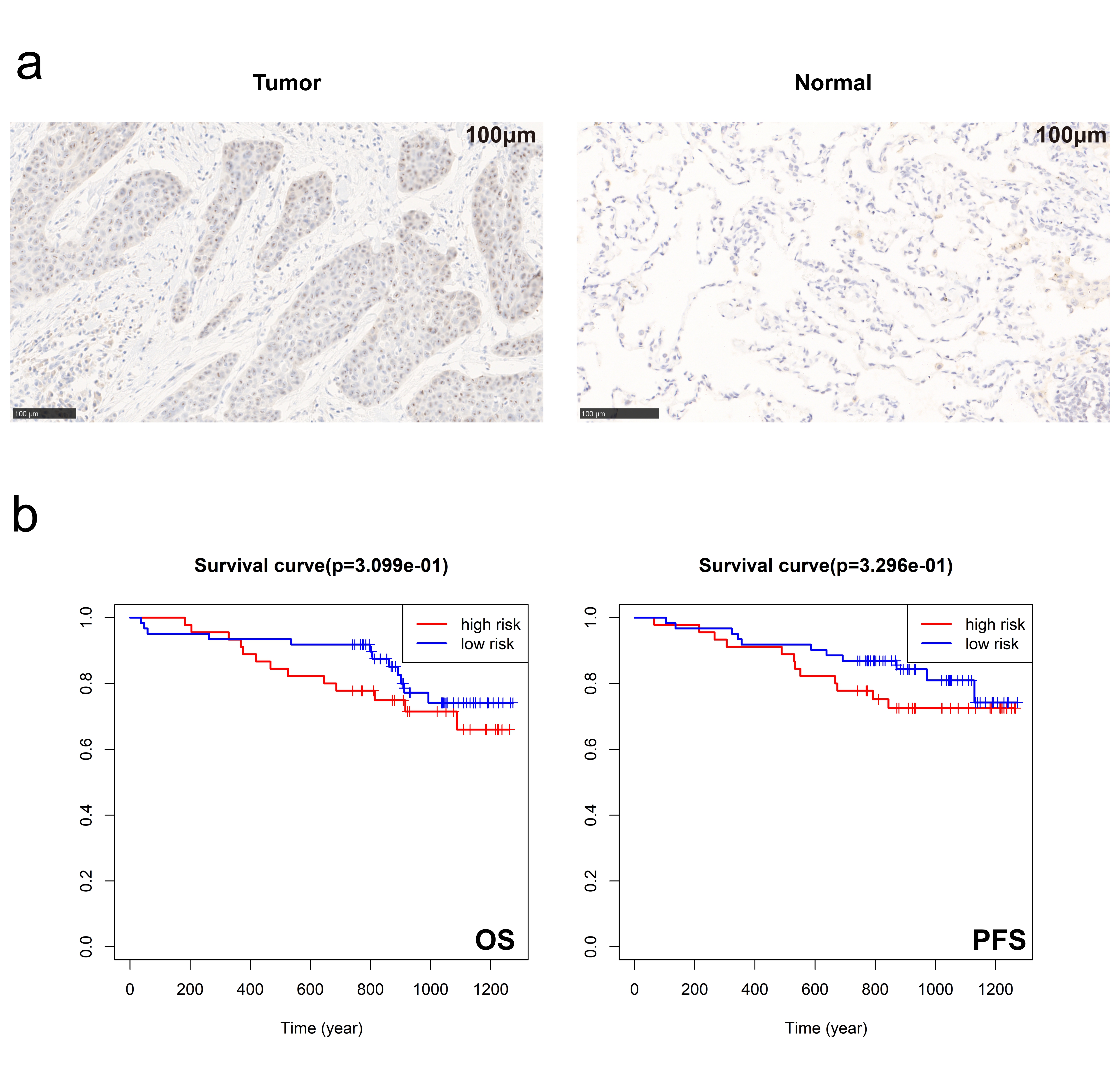

Supplement: Supplementary file 2 [file DataSheet1.ZIP › S1.jpg]

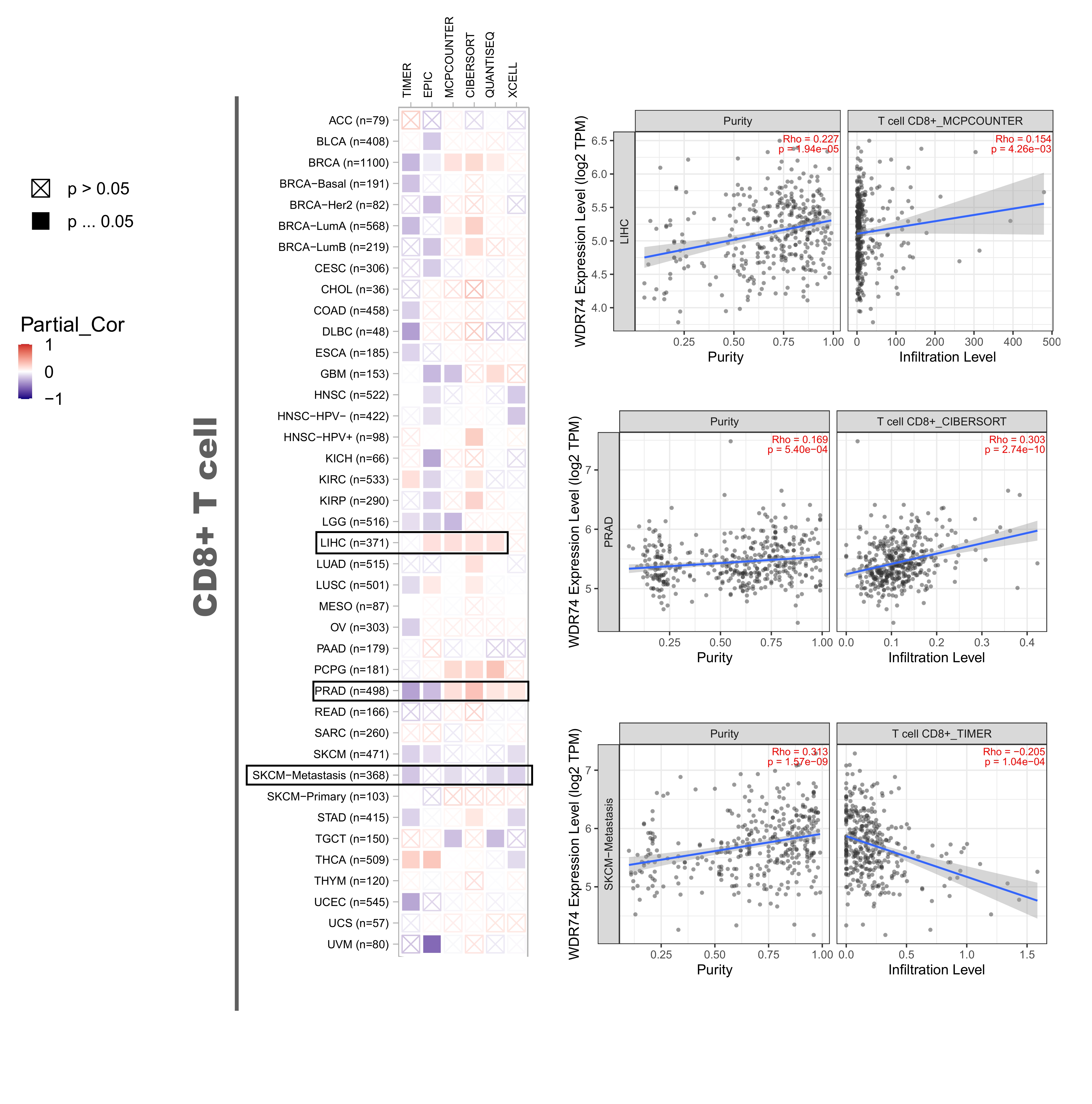

Supplement: Supplementary file 2 [file DataSheet1.ZIP › S10.jpg]

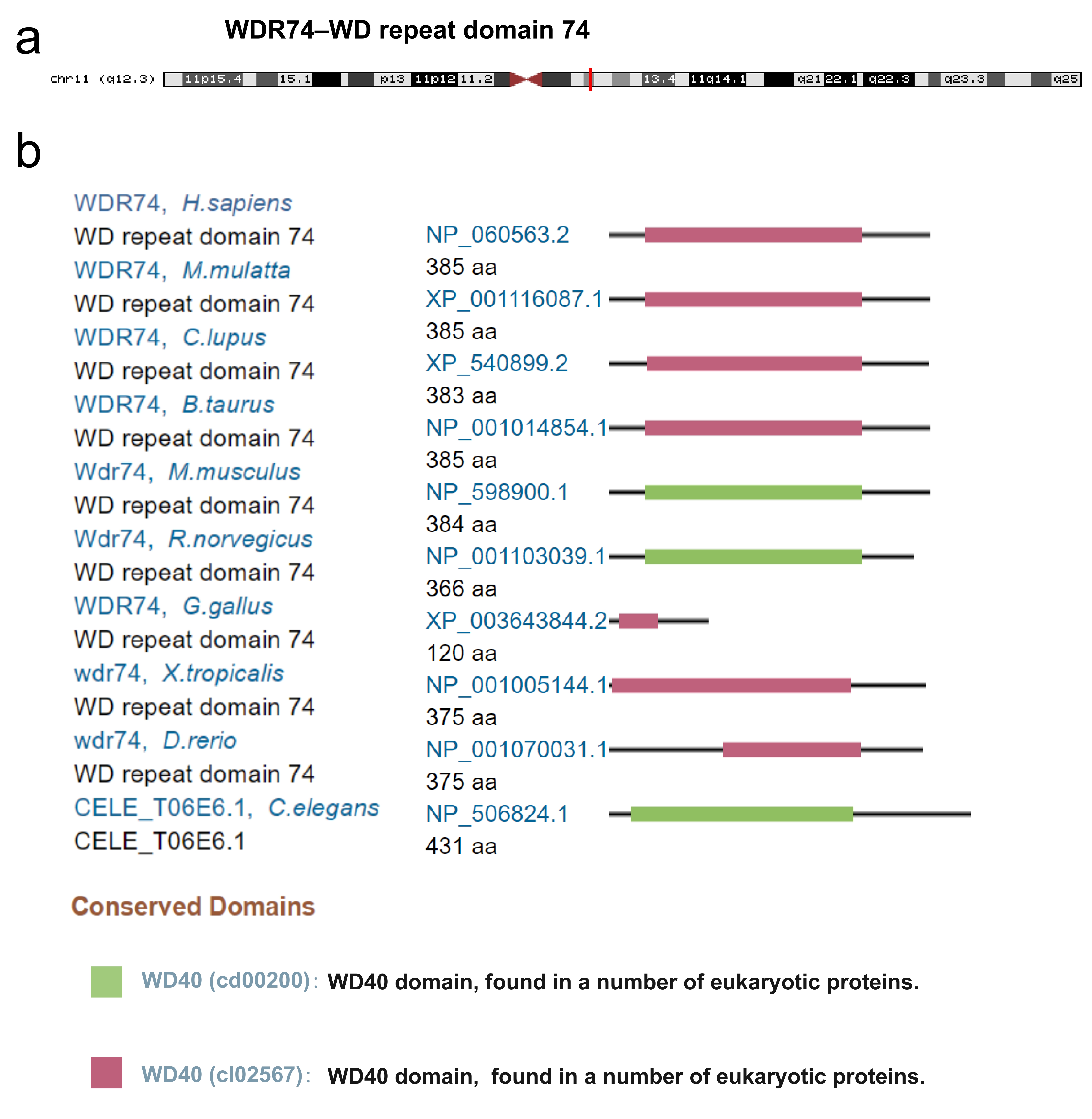

Supplement: Supplementary file 2 [file DataSheet1.ZIP › S2.jpg]

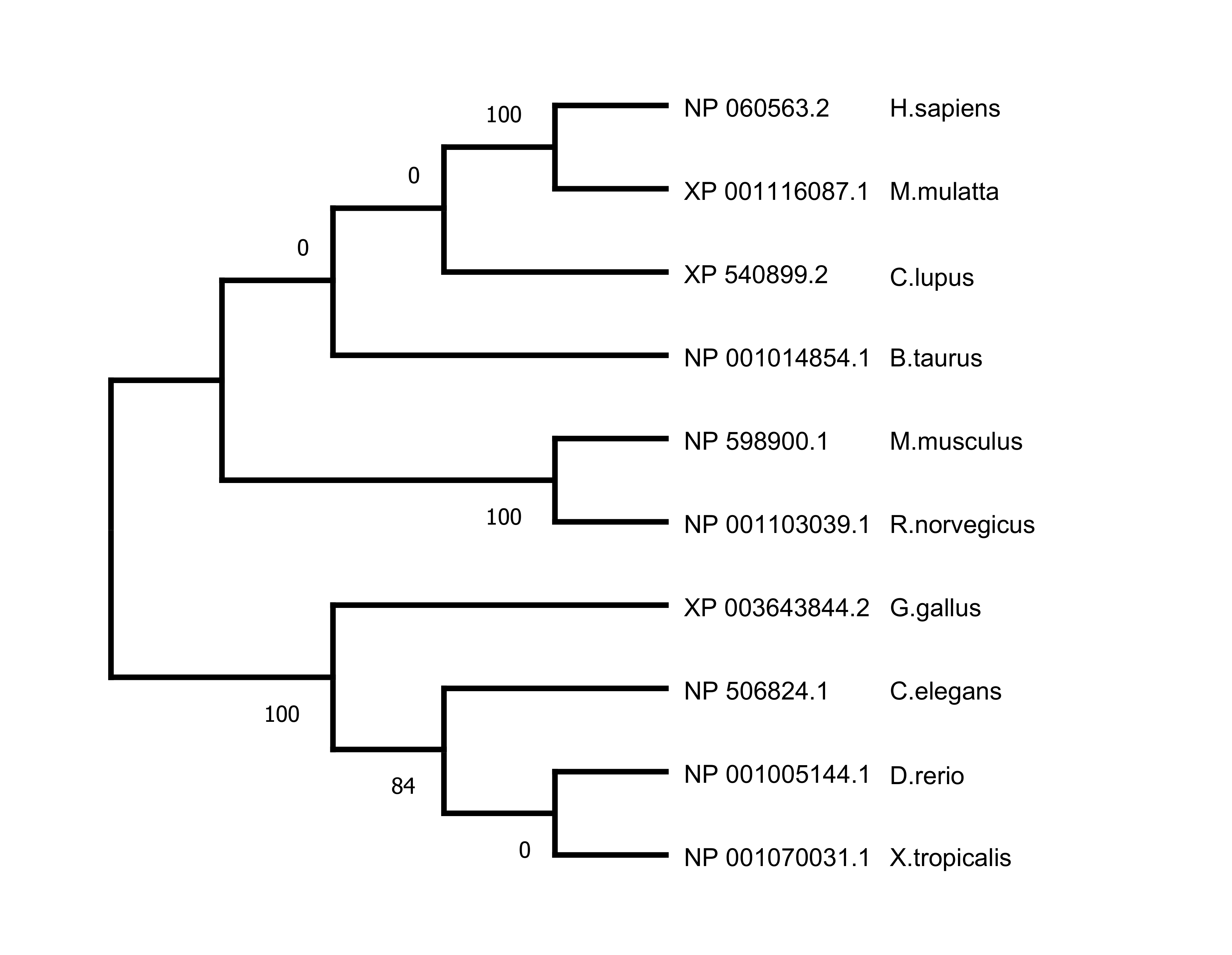

Supplement: Supplementary file 2 [file DataSheet1.ZIP › S3.jpg]

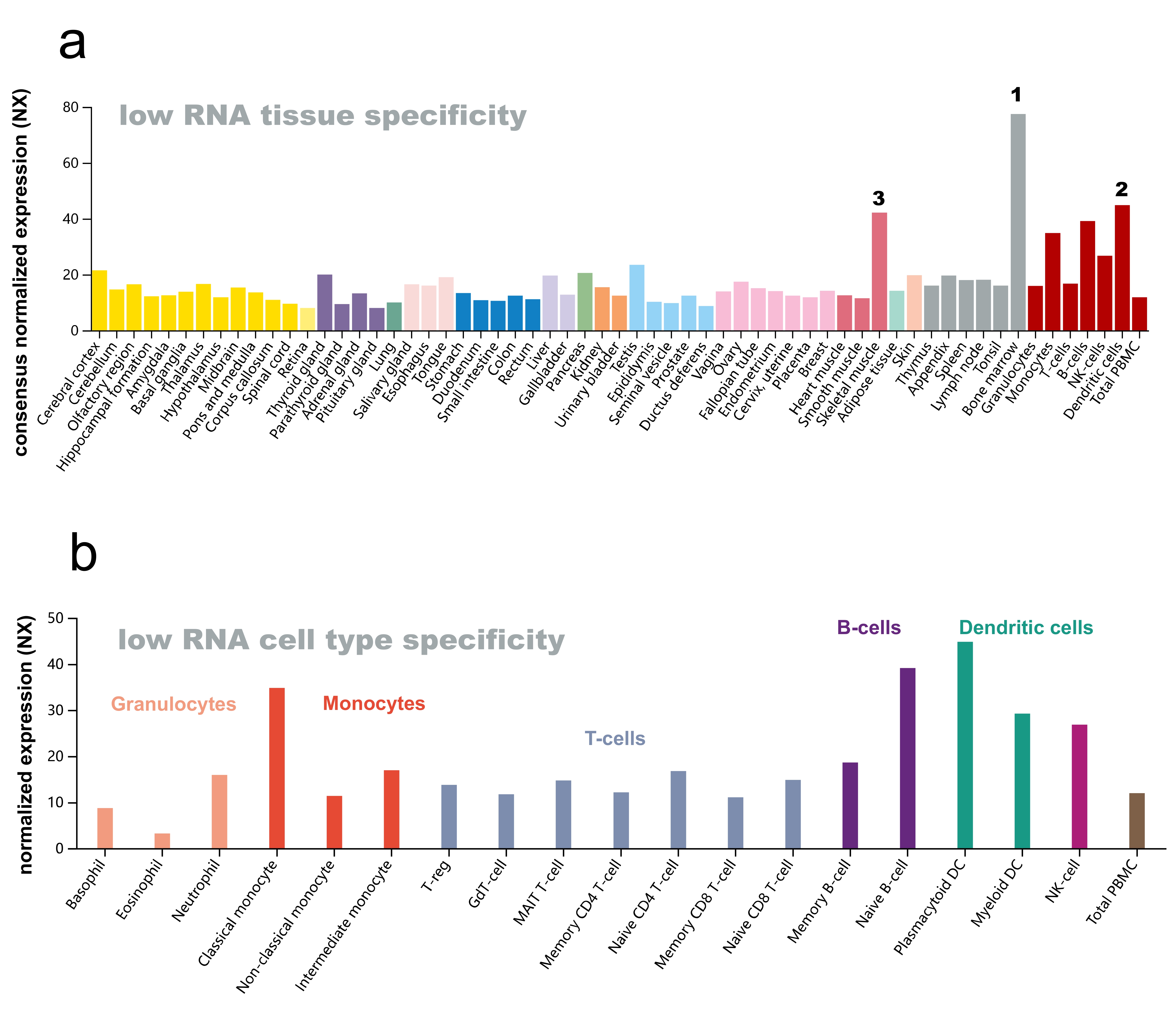

Supplement: Supplementary file 2 [file DataSheet1.ZIP › S4.jpg]

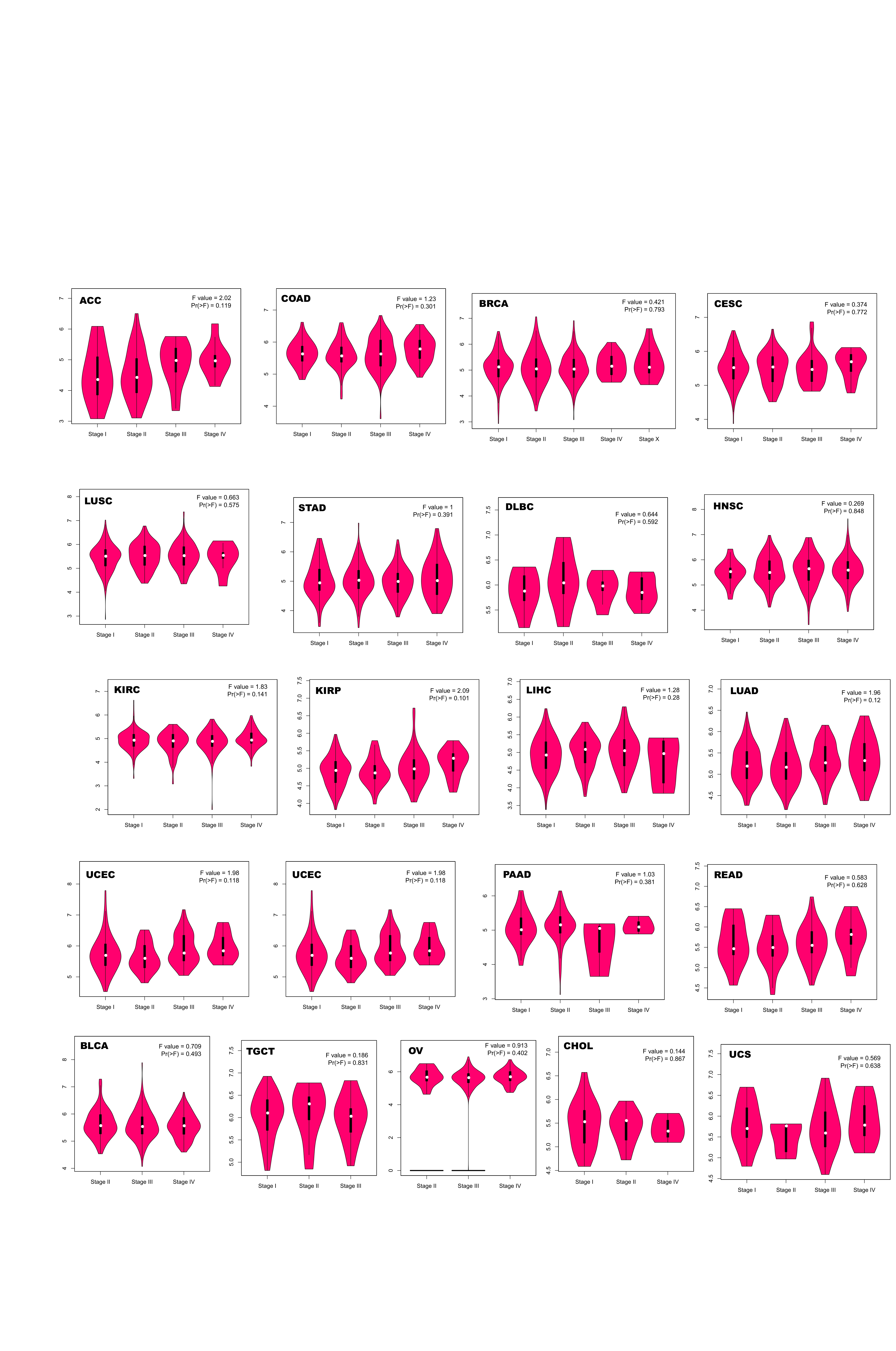

Supplement: Supplementary file 2 [file DataSheet1.ZIP › S5.jpg]

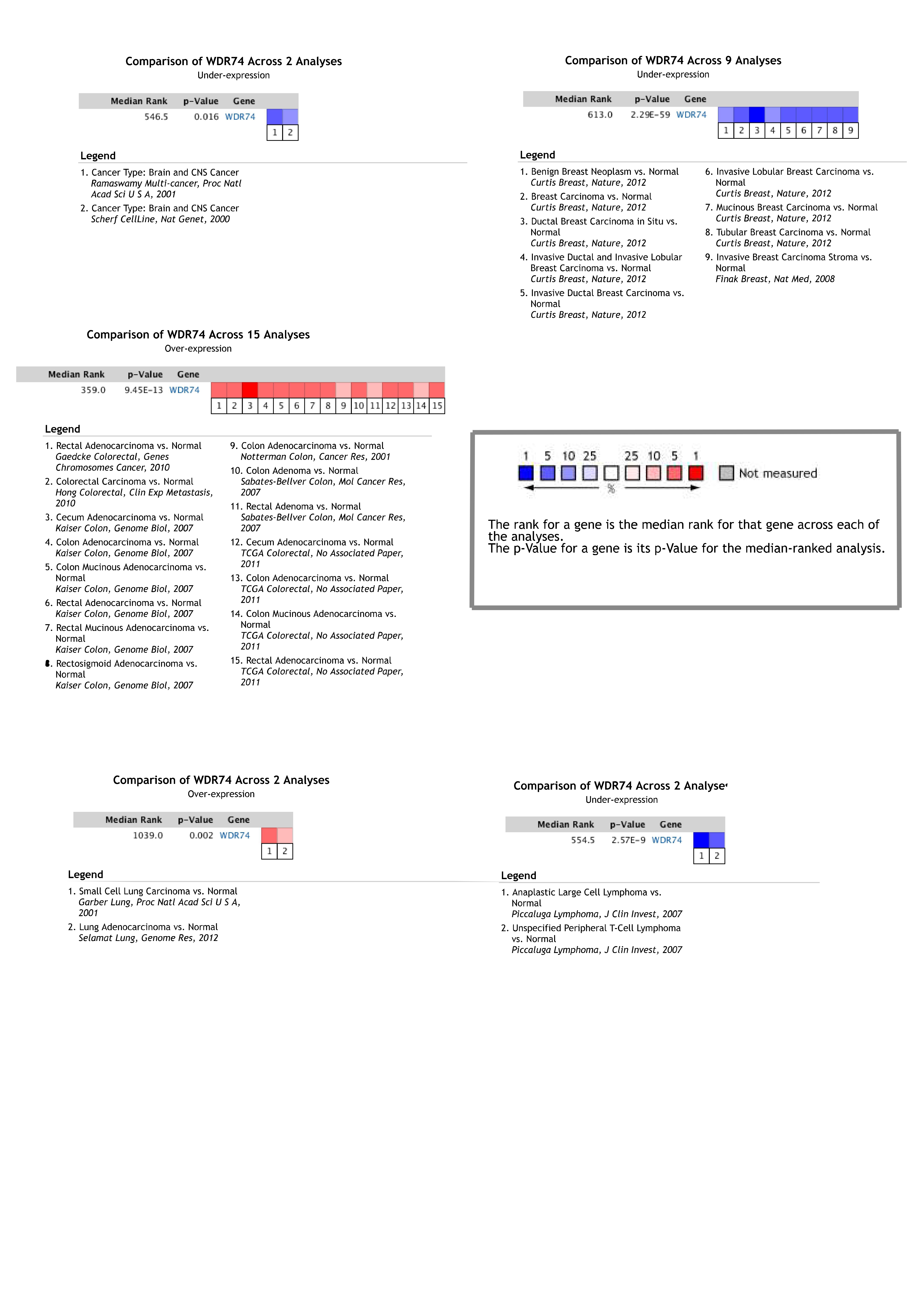

Supplement: Supplementary file 2 [file DataSheet1.ZIP › S6.jpg]

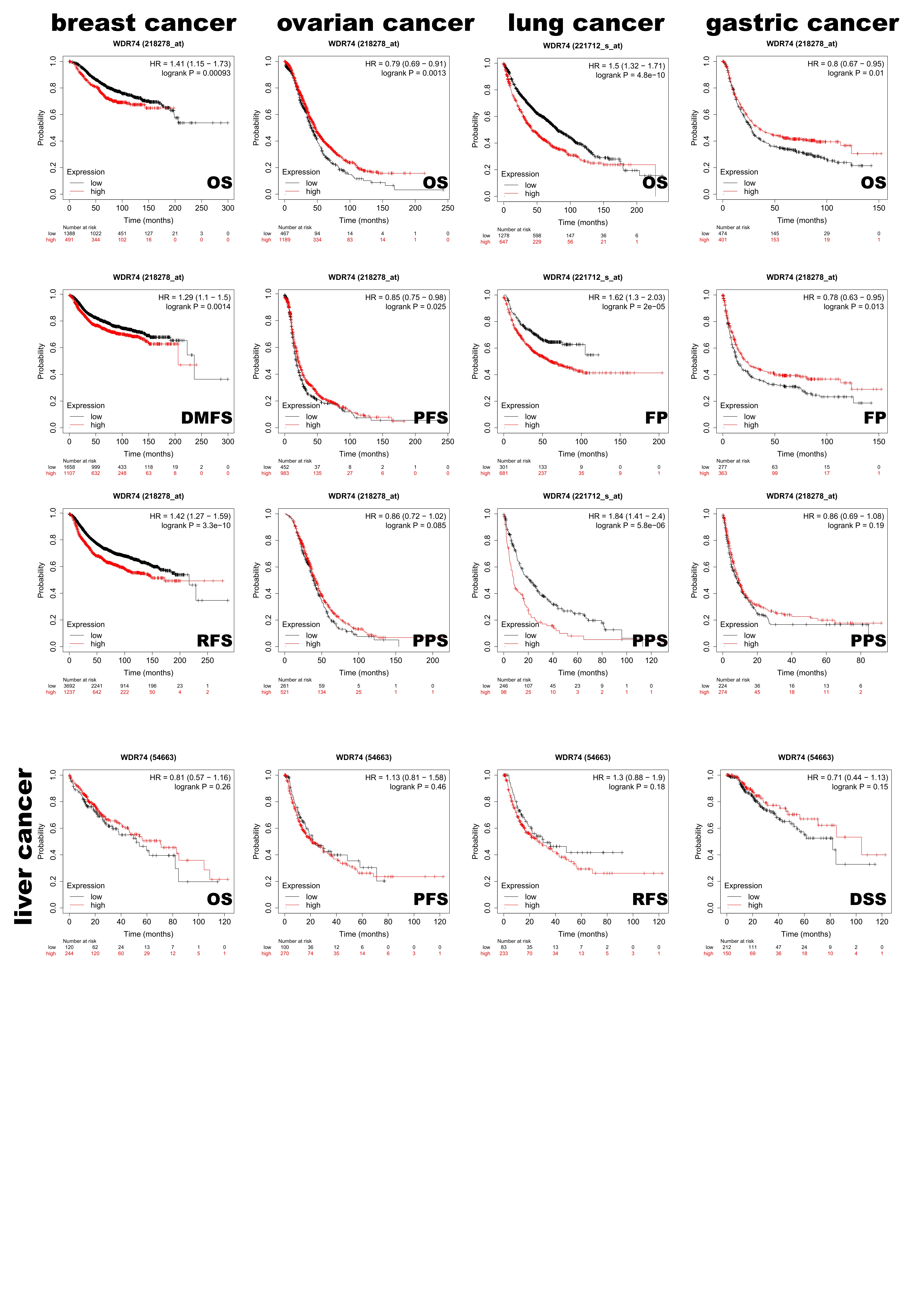

Supplement: Supplementary file 2 [file DataSheet1.ZIP › S7.jpg]

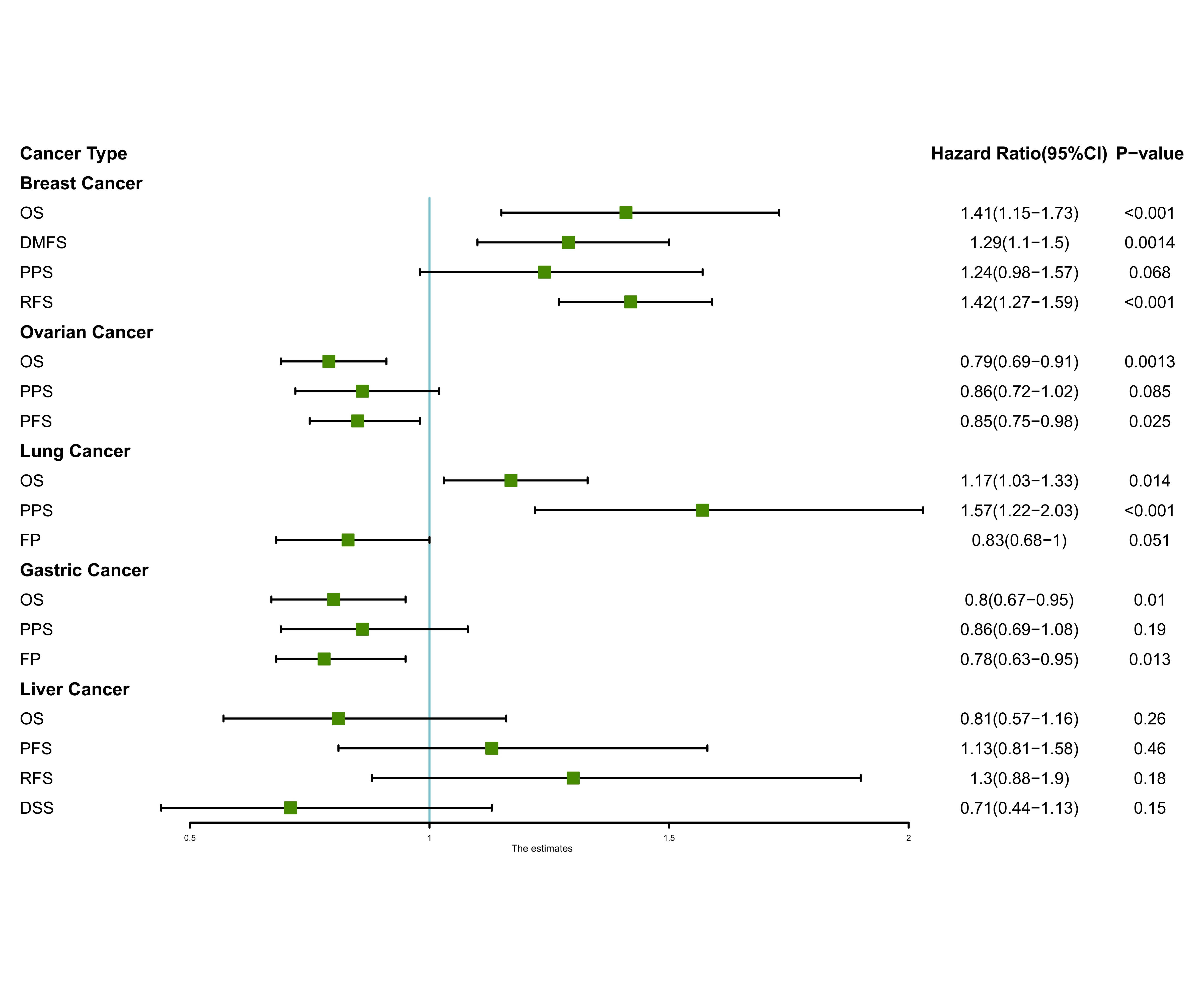

Supplement: Supplementary file 2 [file DataSheet1.ZIP › S8.jpg]

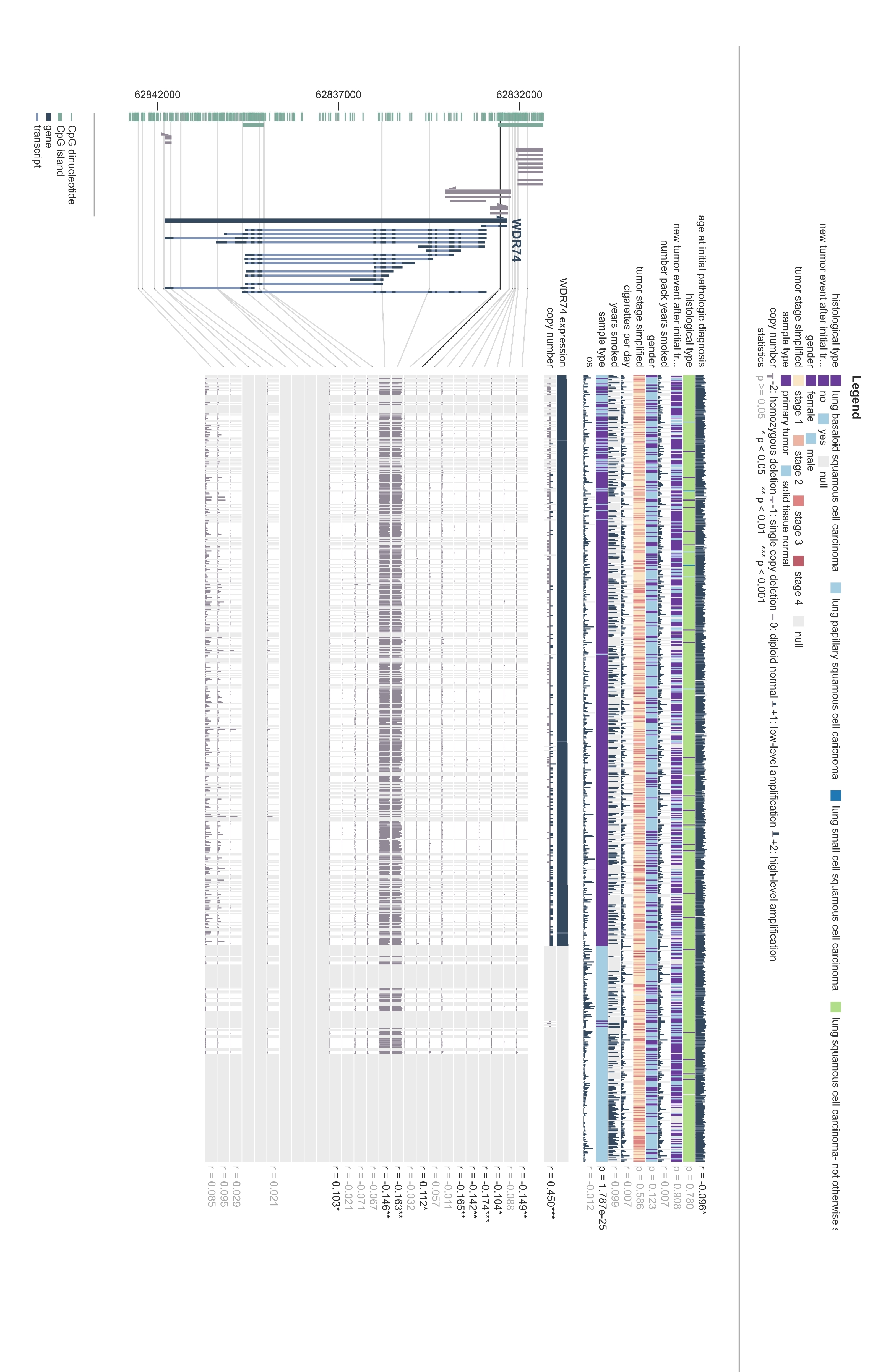

Supplement: Supplementary file 2 [file DataSheet1.ZIP › S9.jpg]
