# Supplementary material for: A Pan-Cancer Analysis of the Oncogenic Role of WD Repeat Domain 74 in Multiple Tumors
Source: Front Genet. 2022 Apr 26;13:860940. doi: 10.3389/fgene.2022.860940 (PMC9086290; doi:10.3389/fgene.2022.860940)
Supplement: Supplementary file 3 [file DataSheet2.docx]

**Table S1. Subgroup analysis on the correlation of *WDR74* expression and prognosis of breast cancer cases.**

| **Factor** | **Subgroup** | **Sample size** | **OS** | | **RFS** | | **DMFS** | | **PPS** | |
| --- | --- | --- | --- | --- | --- | --- | --- | --- | --- | --- |
|  |  |  | **HR** | ***P*** | **HR** | ***P*** | **HR** | ***P*** | **HR** | ***P*** |
| **ER status** | ER positive | 3499 | 1.78 | 0.00032 | 1.29 | 0.0013 | 1.72 | 0.00016 | 1.75 | 0.0026 |
|  | ER negative | 2168 | 1.41 | 0.057 | 0.75 | 0.0063 | 0.81 | 0.14 | 0.73 | 0.23 |
| **TP53 status** | mutated | 272 | 0.5 | 0.11 | 1.45 | 0.14 | 0.55 | 0.12 | 0.29 | 0.01 |
|  | Wild type | 388 | 1.71 | 0.087 | 1.35 | 0.17 | 0.53 | 0.09 | 2.04 | 0.042 |
| **PR status** | PR positive | 1559 | 0.46 | 0.039 | 1.67 | 0.00049 | 1.49 | 0.1 | 0.29 | 0.019 |
|  | PR negative | 1989 | 1.55 | 0.085 | 1.18 | 0.17 | 1.32 | 0.059 | 0.57 | 0.26 |
| **HER2 status** | HER2 positive | 1273 | 1.28 | 0.22 | 1.3 | 0.032 | 0.72 | 0.048 | 1.22 | 0.42 |
|  | HER2 negative | 6262 | 1.58 | 0.00017 | 1.45 | 4e-9 | 1.46 | 1.8e-5 | 1.35 | 0.034 |
| **Grade** | Grade 1 | 576 | 0.54 | 0.18 | 1.66 | 0.048 | 1.9 | 0.11 | 0.26 | 0.0096 |
|  | Grade 2 | 1795 | 1.71 | 0.01 | 0.84 | 0.12 | 1.31 | 0.07 | 2.1 | 0.0012 |
|  | Grade 3 | 2058 | 1.45 | 0.019 | 1.22 | 0.038 | 1.16 | 0.25 | 1.41 | 0.085 |
| **Intrinsic subtype** | Basal | 1494 | 1.51 | 0.031 | 1.14 | 0.25 | 1.27 | 0.14 | 2.53 | 0.0021 |
|  | Luminal A | 3511 | 1.45 | 0.028 | 1.35 | 0.00052 | 1.53 | 0.0022 | 0.76 | 0.18 |
|  | Luminal B | 2015 | 1.33 | 0.14 | 1.32 | 0.0045 | 0.83 | 0.19 | 0.7 | 0.1 |
|  | HER2+ | 515 | 0.53 | 0.04 | 0.42 | 0.00039 | 0.57 | 0.045 | 1.59 | 0.27 |
| **Lymph node status** | Lymph node positive | 2153 | 1.64 | 0.0031 | 1.46 | 1.2e-5 | 1.76 | 7.5e-6 | 1.24 | 0.31 |
|  | Lymph node negative | 2829 | 1.15 | 0.43 | 0.88 | 0.13 | 1.23 | 0.099 | 1.31 | 0.23 |
| **Pietenpol subtype** | Basal-like 1 | 418 | 1.85 | 0.12 | 1.38 | 0.14 | 0.65 | 0.15 | 3.45 | 0.047 |
|  | Basal-like 2 | 165 | 0.39 | 0.074 | 2.11 | 0.014 | 1.5 | 0.31 | NA | NA |
|  | immunomodulatory | 462 | 0.42 | 0.028 | 0.69 | 0.14 | 0.52 | 0.085 | 0.52 | 0.29 |
|  | Mesenchymal | 382 | 0.65 | 0.2 | 0.67 | 0.048 | 0.46 | 0.056 | 0.5 | 0.14 |
|  | Mesenchymal stem-like | 201 | 2.17 | 0.3 | 1.72 | 0.26 | 2.89 | 0.057 | NA | NA |
|  | Luminal androgen receptor | 413 | 1.82 | 0.063 | 1.87 | 0.0021 | 0.61 | 0.096 | 0.64 | 0.32 |

HR, hazard ratio; OS, overall survival; RFS, relapse free survival; DMFS, distant metastasis free survival;

ER, Estrogen receptor; PR, Progesterone receptor; HER2, human epidermal growth factor receptor-2;

TP53, Tumor Protein P53; NA, not available data; *P* value less than 0.05 is shown in bold.

**Table S2. Subgroup analysis on the correlation of *WDR74* expression and prognosis of lung cancer cases.**

| **Factor** | **Subgroup** | **Sample size** | **OS** | | **FP** | | **PPS** | |
| --- | --- | --- | --- | --- | --- | --- | --- | --- |
|  |  |  | **HR** | ***P*** | **HR** | ***P*** | **HR** | ***P*** |
| **Histology** | adenocarcinoma | 865 | 1.14 | 0.27 | 0.86 | 0.35 | 2.07 | 0.0044 |
|  | squamous cell carcinoma | 675 | 1.33 | 0.027 | 0.59 | 0.043 | 0.75 | 0.61 |
| **gender** | female | 817 | 1.28 | 0.056 | 0.72 | 0.035 | 1.71 | 0.0057 |
|  | male | 1387 | 1.14 | 0.096 | 0.84 | 0.2 | 1.56 | 0.017 |
| **smoking history** | exclude those never smoked | 970 | 0.86 | 0.15 | 0.81 | 0.1 | 1.47 | 0.0084 |
|  | only those never smoked | 247 | 0.79 | 0.4 | 1.37 | 0.22 | 2.02 | 0.032 |
| **stage** | stage I | 652 | 1.62 | 0.0016 | 0.73 | 0.15 | 2.77 | 0.0019 |
|  | stage II | 320 | 1.24 | 0.25 | 0.7 | 0.17 | 1.57 | 0.19 |
|  | stage III | 70 | 0.53 | 0.035 | NA | NA | NA | NA |
| **grade** | grade I | 202 | 1.29 | 0.2 | 1.6 | 0.04 | 2 | 0.0057 |
|  | grade II | 310 | 1.45 | 0.031 | 1.35 | 0.16 | 1.63 | 0.071 |
|  | grade III | 77 | 2.41 | 0.036 | 0.44 | 0.064 | 3.25 | 0.057 |
| **AJCC stage t** | t1 | 475 | 1.31 | 0.065 | 0.62 | 0.12 | 2.71 | 0.0029 |
|  | t2 | 686 | 1.13 | 0.28 | 0.75 | 0.06 | 1.62 | 0.0063 |
|  | t3 | 99 | 1.46 | 0.14 | 0.57 | 0.27 | NA | NA |
|  | t4 | 48 | 2.27 | 0.022 | NA | NA | NA | NA |
| **AJCC stage n** | n0 | 863 | 1.28 | 0.022 | 0.78 | 0.14 | 2.3 | 2.3e-5 |
|  | n1 | 296 | 1.21 | 0.28 | 0.58 | 0.025 | 1.86 | 0.039 |
|  | n2 | 113 | 0.85 | 0.42 | 1.93 | 0.17 | 0.55 | 0.095 |
| **AJCC stage m** | m0 | 818 | 1.26 | 0.027 | 1.38 | 0.24 | 2.48 | 0.0032 |
| **surgery** | only surgical margins negative | 730 | 0.81 | 0.075 | 1.15 | 0.3 | 1.55 | 0.0045 |
| **radiotherapy** | no | 276 | 1.2 | 0.37 | 1.31 | 0.23 | 1.83 | 0.0098 |
|  | yes | 73 | 1.45 | 0.19 | 1.95 | 0.028 | 0.57 | 0.056 |
| **chemotherapy** | no | 317 | 1.32 | 0.12 | 0.77 | 0.28 | 1.87 | 0.012 |
|  | yes | 178 | 1.73 | 0.017 | 1.58 | 0.049 | 0.84 | 0.46 |

HR, hazard ratio; AJCC，American Joint Committee on Cancer; OS, overall survival; FP, first progression; PPS, post progression survival; NA, not available data; *P* value less than 0.05 is shown in bold.

| **Factor** | **Subgroup** | **Sample size** | **OS** | | **PFS** | | **PPS** | |  |
| --- | --- | --- | --- | --- | --- | --- | --- | --- | --- |
|  |  |  | **HR** | ***p*** | **HR** | ***p*** | **HR** | ***p*** | |
| **Histology** | Endometrioid | 62 | 0.41 | 0.41 | 4.59 | 0.025 | NA | NA | |
|  | Serous | 1232 | 0.78 | 0.002 | NA | NA | 0.85 | 0.082 | |
| **Stage** | Stage 1 | 107 | 1.92 | 0.26 | 2.63 | 0.09 | NA | NA | |
|  | Stage 2 | 72 | 0.43 | 0.14 | 1.66 | 0.15 | 0.28 | 0.049 | |
|  | Stage 3 | 1079 | 0.77 | 0.0028 | 0.9 | 0.2 | 0.89 | 0.23 | |
|  | Stage 4 | 189 | 0.81 | 0.32 | 1.46 | 0.045 | 1.38 | 0.19 | |
| **Grade** | Grade 1 | 56 | 0.43 | 0.13 | 0.65 | 0.46 | NA | NA | |
|  | Grade 2 | 325 | 0.58 | 0.00081 | 0.74 | 0.058 | 0.7 | 0.068 | |
|  | Grade 3 | 1024 | 0.81 | 0.013 | 0.89 | 0.19 | 0.85 | 0.12 | |
|  | Grade 4 | 21 | 2.15 | 0.13 | NA | NA | NA | NA | |
| **TP53 mutation** | Mutated | 516 | 1.11 | 0.37 | 1.14 | 0.25 | 1.34 | 0.035 | |
|  | Wild type | 102 | 0.61 | 0.12 | 1.91 | 0.017 | 0.56 | 0.074 | |
| **Debulk** | optimal | 802 | 0.57 | 4.4e-7 | 0.7 | 0.00043 | 0.58 | 0.00016 | |
|  | suboptimal | 536 | 0.81 | 0.043 | 0.9 | 0.34 | 1.22 | 0.13 | |
| **Chemotherapy** | Contains platin | 1438 | 0.81 | 0.0085 | 0.86 | 0.024 | 0.86 | 0.11 | |
|  | Contains Taxol | 821 | 0.74 | 0.003 | 0.83 | 0.036 | 0.83 | 0.073 | |
|  | Contains Taxol+platin | 804 | 0.73 | 0.0025 | 0.84 | 0.057 | 0.83 | 0.087 | |
|  | Contains Avastin | 50 | 0.21 | 0.0039 | 0.42 | 0.017 | 0.22 | 0.0054 | |
|  | Contains Docetaxel | 108 | 0.67 | 0.079 | 0.64 | 0.11 | 0.76 | 0.42 | |
|  | Contains Gemcitabine | 135 | 0.57 | 0.051 | 0.63 | 0.034 | 0.59 | 0.035 | |
|  | Contains Paclitaxel | 248 | 0.46 | 0.0013 | 0.75 | 0.12 | 0.48 | 0.0071 | |
|  | Contains Topotecan | 119 | 0.8 | 0.31 | 0.65 | 0.054 | 0.78 | 0.28 | |

**Table S3. Subgroup analysis on the correlation of *WDR74* expression and prognosis of ovarian cancer cases.**

HR, hazard ratio; OS, overall survival; PFS, progress free survival; PPS, post progression survival;

TP53, Tumor Protein P53; NA, not available data; *P* value less than 0.05 is shown in bold.

**Table S4. Subgroup analysis on the correlation of *WDR74* expression and prognosis of gastric cancer cases.**

| **Factor** | **Subgroup** | **Sample size** | **OS** | | **FP** | | **PPS** | |
| --- | --- | --- | --- | --- | --- | --- | --- | --- |
|  |  |  | **HR** | ***P*** | **HR** | ***P*** | **HR** | ***P*** |
| **Gender** | Female | 244 | 0.62 | 0.0092 | 0.6 | 0.0069 | 0.68 | 0.088 |
|  | Male | 566 | 0.85 | 0.13 | 0.85 | 0.17 | 0.85 | 0.2 |
| **Stage** | Stage 1 | 69 | 0.43 | 0.14 | 0.46 | 0.19 | 2.99 | 0.29 |
|  | Stage 2 | 145 | 1.61 | 0.19 | 1.67 | 0.15 | 0.46 | 0.08 |
|  | Stage 3 | 319 | 0.87 | 0.41 | 1.25 | 0.25 | 1.39 | 0.13 |
|  | Stage 4 | 152 | 1.39 | 0.092 | 1.32 | 0.18 | 0.73 | 0.2 |
| **Stage t** | t2 | 253 | 1.5 | 0.13 | 1.56 | 0.08 | 0.65 | 0.12 |
|  | t3 | 208 | 1.46 | 0.034 | 1.28 | 0.15 | 1.94 | 0.0019 |
|  | t4 | 39 | 0.42 | 0.11 | 0.59 | 0.18 | 0.51 | 0.29 |
| **Stage n** | n0 | 76 | 0.43 | 0.082 | 0.4 | 0.062 | 0.2 | 0.087 |
|  | n1 | 232 | 0.78 | 0.26 | 1.32 | 0.17 | 1.34 | 0.21 |
|  | n2 | 129 | 1.5 | 0.078 | 1.28 | 0.27 | 1.27 | 0.33 |
|  | n3 | 76 | 1.32 | 0.36 | 0.61 | 0.13 | 0.6 | 0.1 |
| **Stage m** | m0 | 459 | 0.81 | 0.15 | 0.79 | 0.088 | 0.87 | 0.36 |
|  | m1 | 58 | 2.25 | 0.0079 | 2.74 | 0.0016 | 0.81 | 0.57 |
| **HER2** | negative | 641 | 0.73 | 0.012 | 0.78 | 0.076 | 0.76 | 0.11 |
|  | positive | 424 | 0.58 | 6.9e-05 | 0.57 | 0.00053 | 0.67 | 0.04 |
| **Lauren classification** | Instestinal | 336 | 0.81 | 0.25 | 1.46 | 0.034 | 1.65 | 0.061 |
|  | Diffuse | 248 | 0.84 | 0.31 | 0.84 | 0.31 | 0.76 | 0.16 |
|  | Mixed | 33 | 3.41 | 0.089 | 0.6 | 0.37 | NA | NA |
| **Differentiation** | Poorly | 166 | 1.48 | 0.06 | 1.7 | 0.026 | 2.05 | 0.038 |
|  | Moderately | 67 | 1.47 | 0.25 | 1.62 | 0.13 | 1.99 | 0.14 |
|  | Well | 32 | 0.42 | 0.11 | NA | NA | NA | NA |
| **Treatment** | Surgery alone | 393 | 1.32 | 0.07 | 0.82 | 0.17 | 1.21 | 0.24 |
|  | 5-Fu based adjuvant | 157 | 1.21 | 0.28 | 1.37 | 0.1 | 1.39 | 0.1 |
| **Perforation** | No | 169 | 1.31 | 0.23 | 1.3 | 0.19 | 1.65 | 0.092 |

HR, hazard ratio; OS, overall survival; FP, first progression; PPS, post progression survival;

HER2, human epidermal growth factor receptor-2; NA, not available data; *P* value less than 0.05 is shown in bold.

**Table S5. Subgroup analysis on the correlation of *WDR74* expression and prognosis of liver cancer cases.**

| **Factor** | **Subgroup** | **Sample size** | **OS** | | **PFS** | | **RFS** | | **DSS** | |
| --- | --- | --- | --- | --- | --- | --- | --- | --- | --- | --- |
|  |  |  | **HR** | ***P*** | **HR** | ***P*** | **HR** | ***P*** | **HR** | ***P*** |
| **Stage** | Stage 1 | 171 | 1.59 | 0.15 | 0.84 | 0.54 | 1.3 | 0.37 | 0.45 | 0.071 |
|  | Stage 2 | 86 | 1.6 | 0.27 | 0.74 | 0.37 | 1.81 | 0.086 | 2.67 | 0.12 |
|  | Stage 3 | 85 | 0.6 | 0.14 | 1.56 | 0.14 | 0.65 | 0.18 | 0.52 | 0.14 |
| **Grade** | Grade 1 | 55 | 1.59 | 0.33 | 3.24 | 0.0028 | 4.01 | 0.0032 | 1.77 | 0.46 |
|  | Grade 2 | 177 | 0.73 | 0.23 | 1.32 | 0.23 | 1.49 | 0.11 | 1.41 | 0.33 |
|  | Grade 3 | 122 | 0.64 | 0.21 | 0.48 | 0.0097 | 0.48 | 0.016 | 0.26 | 0.019 |
| **AJCC_T** | T1 | 181 | 1.52 | 0.19 | 0.85 | 0.5 | 1.35 | 0.33 | 0.55 | 0.14 |
|  | T2 | 94 | 0.62 | 0.23 | 0.69 | 0.24 | 1.64 | 0.21 | 1.64 | 0.33 |
|  | T3 | 80 | 0.61 | 0.15 | 1.35 | 0.31 | 0.58 | 0.12 | 0.48 | 0.088 |
| **Gender** | Female | 121 | 0.65 | 0.18 | 0.65 | 0.11 | 1.52 | 0.24 | 0.53 | 0.13 |
|  | Male | 250 | 1.39 | 0.15 | 1.38 | 0.091 | 1.36 | 0.15 | 1.37 | 0.29 |
| **Vascular invasion** | None | 205 | 0.6 | 0.053 | 0.74 | 0.26 | 0.72 | 0.27 | 0.53 | 0.071 |
|  | micro | 93 | 0.42 | 0.039 | 0.7 | 0.22 | 1.72 | 0.1 | 0.44 | 0.14 |
| **Race** | White | 184 | 0.73 | 0.23 | 0.71 | 0.12 | 0.74 | 0.21 | 0.55 | 0.061 |
|  | Asian | 158 | 1.46 | 0.22 | 1.88 | 0.017 | 1.95 | 0.019 | 2.37 | 0.1 |
| **Sorafenib treatment** | treated | 30 | 9.43 | 0.011 | 2.55 | 0.085 | 2.17 | 0.22 | 9.43 | 0.011 |
| **Alcohol consumption** | Yes | 117 | 2.05 | 0.048 | 1.28 | 0.42 | 0.74 | 0.3 | 1.55 | 0.31 |
|  | none | 205 | 0.65 | 0.075 | 1.12 | 0.59 | 1.48 | 0.15 | 0.56 | 0.12 |
| **Hepatitis virus** | Yes | 153 | 0.73 | 0.36 | 1.25 | 0.35 | 1.48 | 0.17 | 0.32 | 0.027 |
|  | none | 169 | 1.53 | 0.12 | 1.27 | 0.36 | 1.63 | 0.13 | 1.78 | 0.1 |

HR, hazard ratio; AJCC，American Joint Committee on Cancer; OS, overall survival; PFS, progress free survival;

RFS, relapse free survival; DSS, disease specific surviva; NA, not available data; *P* value less than 0.05 is shown in bold.

**Table S6. *WDR74* mutation in LUSC**

| **Tumor_Sample_Barcode** | **Chromosome** | **Start_position** | **End_position** | **Strand** | **Reference_Allele** | **Tumor_Seq_Allele2** | **Type** |  |
| --- | --- | --- | --- | --- | --- | --- | --- | --- |
| NCC-01-067 | 11 | 62610449 | 62610449 | - | C | G | Promoter core |  |
| NCC-01-013 | 11 | 62609520 | 62609520 | - | G | C | Promoter core |  |
| NCC-01-080 | 11 | 62609427 | 62609427 | - | G | C | Promoter core |  |
| NCC-01-068 | 11 | 62609329 | 62609329 | - | C | A | Promoter core |  |
| NCC-01-019 | 11 | 62609329 | 62609329 | - | C | T | Promoter core |  |
| NCC-01-018 | 11 | 62609329 | 62609329 | - | C | A | Promoter core |  |
| NCC-01-073 | 11 | 62609327 | 62609327 | - | A | G | Promoter core |  |
| NCC-01-069 | 11 | 62609220 | 62609220 | - | A | G | Promoter core |  |
| NCC-01-005 | 11 | 62609212 | 62609212 | - | G | A | Promoter core |  |
| NCC-01-101 | 11 | 62609195 | 62609195 | - | A | G | Promoter core |  |
| NCC-01-002 | 11 | 62609179 | 62609179 | - | A | T | Promoter core |  |
| NCC-01-080 | 11 | 62609178 | 62609178 | - | A | G | Promoter core |  |
| NCC-01-049 | 11 | 62609178 | 62609178 | - | A | G | Promoter core |  |
| NCC-01-006 | 11 | 62609131 | 62609131 | - | G | T | Promoter core |  |
| NCC-01-054 | 11 | 62609112 | 62609112 | - | A | G | Promoter core |  |
| NCC-01-013 | 11 | 62608945 | 62608945 | - | C | G | Promoter core |  |
